# Somatic mutations in lymphocytes in patients with immune-mediated aplastic anemia

**DOI:** 10.1038/s41375-021-01231-3

**Published:** 2021-03-30

**Authors:** Sofie Lundgren, Mikko A. I. Keränen, Matti Kankainen, Jani Huuhtanen, Gunilla Walldin, Cassandra M. Kerr, Michael Clemente, Freja Ebeling, Hanna Rajala, Oscar Brück, Harri Lähdesmäki, Sari Hannula, Tiina Hannunen, Pekka Ellonen, Neal S. Young, Seishi Ogawa, Jaroslaw P. Maciejewski, Eva Hellström-Lindberg, Satu Mustjoki

**Affiliations:** 1 Hematology Research Unit Helsinki, University of Helsinki and Helsinki University Hospital Comprehensive Cancer Center, Helsinki, Finland; 2 Translational Immunology Research program and Department of Clinical Chemistry and Hematology, University of Helsinki, Helsinki, Finland; 3 Department of Hematology, Helsinki University Hospital Comprehensive Cancer Center, Helsinki, Finland; 4 Medical and Clinical Genetics, University of Helsinki and Helsinki University Hospital, Helsinki, Finland; 5 Department of Computer Science, School of Science, Aalto University, Espoo, Finland; 6 Center for Hematology and Regenerative Medicine, Department of Medicine, Karolinska Institute and Karolinska University Hospital, Stockholm, Sweden; 7 Translational Hematology and Oncology Department, Taussig Cancer Center, Cleveland Clinic, Cleveland, OH USA; 8 Institute of Molecular Medicine Finland, HILIFE, University of Helsinki, Helsinki, Finland; 9 Hematology Branch, National Heart Lung and Blood Institute (NHLBI), National Institutes of Health (NIH), Bethesda, MD USA; 10 Pathology and Tumor Biology, Graduate School of Medicine, Kyoto University, Kyoto, Japan; 11 ICAN Digital Precision Cancer Medicine Flagship, Helsinki, Finland

**Keywords:** Autoimmune diseases, Genetics research, Anaemia

## Abstract

The prevalence and functional impact of somatic mutations in nonleukemic T cells is not well characterized, although clonal T-cell expansions are common. In immune-mediated aplastic anemia (AA), cytotoxic T-cell expansions are shown to participate in disease pathogenesis. We investigated the mutation profiles of T cells in AA by a custom panel of 2533 genes. We sequenced CD4+ and CD8+ T cells of 24 AA patients and compared the results to 20 healthy controls and whole-exome sequencing of 37 patients with AA. Somatic variants were common both in patients and healthy controls but enriched to AA patients’ CD8+ T cells, which accumulated most mutations on JAK-STAT and MAPK pathways. Mutation burden was associated with CD8+ T-cell clonality, assessed by T-cell receptor beta sequencing. To understand the effect of mutations, we performed single-cell sequencing of AA patients carrying *STAT3* or other mutations in CD8+ T cells. *STAT3* mutated clone was cytotoxic, clearly distinguishable from other CD8+ T cells, and attenuated by successful immunosuppressive treatment. Our results suggest that somatic mutations in T cells are common, associate with clonality, and can alter T-cell phenotype, warranting further investigation of their role in the pathogenesis of AA.

## Introduction

Acquired aplastic anemia (AA) is an immune-mediated bone marrow failure syndrome in which cytotoxic T cells mediate the destruction of hematopoietic stem and progenitor cells (HSPCs) [1–3]. Characteristic features of immune-mediated AA are clonal alterations in HSPCs: X-chromosome skewing [4], cytogenetic abnormalities [5, 6], uniparental disomy of the 6p [7], somatic phosphatidylinositol glycan class A gene mutations leading to paroxysmal nocturnal hemoglobinuria [8], and somatic mutations in genes related to clonal hematopoiesis (CH) [9–14]. It has been suggested that some clonal changes in target cells are enriched by selective pressure from autoreactive T cells, and hence they may serve as an immune escape mechanism [1, 15].

Oligoclonal CD8+ T cells are frequently detected in AA and they are able to induce apoptosis of autologous HSPC [16, 17]. Furthermore, T cells in AA patients are known to be aberrantly active [18–20], but their antigenic target on HSPC remains unknown.

Previously, we and others have detected somatic mutations in T cells in patients with various immune-mediated disorders: newly diagnosed rheumatoid arthritis [21], Felty’s syndrome [22], pure red cell aplasia [23], multiple sclerosis [24, 25], chronic graft-versus-host disease [26], and T-cell large granular lymphocytic (T-LGL) leukemia [27–29]. In T-LGL leukemia and Felty’s syndrome activating *STAT3* mutations in CD8+ T cells are the hallmark of the disease, but *STAT3* mutations have also been discovered in 11% of AA patients [30].

Although the role of somatic events in lymphocytes is not known in detail in patients with autoimmune diseases, they may alter the lymphocyte phenotype and function, and in that manner contribute to the pathogenesis. Mutations may either arise de novo in mature lymphocytes during cell division or mutations of CH may drift to lymphocytes, which is a phenomenon called lympho-myeloid CH [31, 32]. In the current study, we characterized the spectrum of somatic mutations in T cells in patients with AA. Furthermore, we evaluated their origins and described their potential impact on single-cell resolution at the diagnosis, relapse, and the recovery of AA.

We show that somatic mutations in T cells are common in AA and even in healthy individuals. These findings extend our understanding of the presence of somatic mutations in nonmalignant diseases and provide basis for the further development of novel therapeutic and diagnostic approaches in lymphocyte-mediated autoimmune diseases.

## Methods

### Samples analyzed by immunogene panel sequencing

Peripheral blood and bone marrow were collected from patients suffering from immune-mediated AA (*n* = 24), see Table S1 for clinical characteristics of the cohort. Patients were recruited from the Department of Hematology in the Helsinki University Hospital and from other participating university hospitals (Karolinska Institute, Sweden, and Cleveland Clinic, Cleveland, OH, USA). This study was approved by the local Ethics Committees, and the principles of Helsinki Declaration were followed. All patients had given their written informed consent before sample collection. Age-matched healthy blood donor buffy coats were provided by Finnish Red Cross Blood Service (*n* = 20).

### Diagnosis of AA

The diagnosis of immune-mediated AA was based on hypocellular (cellularity < 30%) bone marrow and exclusion of constitutional genetic defects and chemical and physiological damage. The severity was based on Camitta’s criteria [33]. Moderate AA was defined as decreased bone marrow cellularity and cytopenias not qualifying for severe AA and exclusion of other causes.

### Sample preparation and immunogene panel sequencing

Sample preparation and immunogene panel sequencing of CD4+ and CD8+ T cells was performed as previously described [34]. A custom gene panel based on 2533 candidate genes along pathways important in innate and adaptive immunity was used. See Supplementary Methods for further information. The sequencing was performed at the Institute for Molecular Medicine Finland, HiLIFE.

### Other genomic datasets

We analyzed raw whole-exome sequencing (WES) data from a previously published dataset of 37 AA patients from Yoshizato et al. [13]. In our analysis, we included CD3− and CD3+ sample from each patient. We also utilized raw WES data from skin samples of nine AA patients, sequenced as described previously [27]. WES data from skin samples were used as a part of panel of normals (PON) for filtering variants detected with WES and immunogene panel sequencing (see Supplementary Methods).

All cohorts used in somatic variant analysis are summarized in Table 1 and study design is illustrated in Fig. S1.

**Table 1:** Patient cohorts in somatic variant analysis.

Dataset	Number of subjects	Purpose
Immunogene panel sequencing of immune-mediated AA patients	24	To investigate somatic mutations in T cells
Immunogene panel sequencing of healthy individuals	20	PON for immunogene panel samples and comparison of adjusted mutation burden for AA samples
Skin WES of patients with AA	9	PON for WES samples and immunogene panel samples
CD3+ and CD3− MNC WES of patients with AA [13]	37	To compare CH derived mutations with somatic mutations seen in T cells

*AA* aplastic anemia, *PON* panel of normals, *WES* whole-exome sequencing, *MNC* mononuclear cell, *CH* clonal hematopoiesis.

### Identification and analysis of potentially pathogenic somatic variants

All datasets were processed and analyzed according to a previously described GATK practice [35], see Supplementary Methods for more detailed description. Following this processing, a joint recalling of newly detected variants and known hotspot variants (listed in Table S2) across all samples was done by running MuTect2 in genotyping mode. Variants detected in both CD4+ and CD8+ T cells of same patient were interpreted as lymphoid precursor (LP) variants, and the rest of the variants were categorized as CD8+ and CD4+ specific variants (list of detected variants in Table S3). Adjusted somatic mutation burden and mutational signatures were calculated and variant effects were predicted as described in Supplementary Methods.

### Amplicon validation

Amplicon sequencing was performed to validate a selected set of mutations found in the immunogene panel sequencing (Table S4). Details of amplicon sequencing and analysis are provided in Supplementary Methods.

### Analysis of clonal T-cell populations

Sorted CD8+ T-cell fractions of available samples were analyzed with a multiplexed PCR assay that targets the variable CDR3 region of the rearranged TCRβ locus [36]. Next-generation sequencing was performed, and data were analyzed with the ImmunoSEQ analysis tools provided by Adaptive Biotechnologies. Clonality was calculated as 1 − Shannon’s entropy for all rearrangements [37]. TCR Vβ family based flow cytometry and sorting are described in Supplementary Methods.

### Single-cell gene expression and V(D)J transcript profiling and data analysis

Frozen MNC from PB or BM were sorted with BD Influx Cell sorter and the gene and V(D)J transcript profiles were studied with 10X Genomics Chromium Single Cell V(D)J and 5′ Gene Expression platform. A detailed description of sample processing and sequencing is provided in Supplementary Methods and Fig. S2.

Detailed description of bioinformatic analyses is provided in  Supplementary Methods and Figs. S3–S5. Tables S5 and S6 contain lists of differentially expressed genes used in cluster annotation.

### Statistical testing

Due to non-Gaussian data distributions, nonparametric tests were used, including Mann–Whitney *U* test in comparisons between two groups, Kruskal–Wallis in comparisons between three or more groups and Spearman’s rank test in correlation with R (3.6.0).

## Results

### Clonal CD8+ T-cell populations are associated with increased somatic mutation burden

**Fig. 1:**
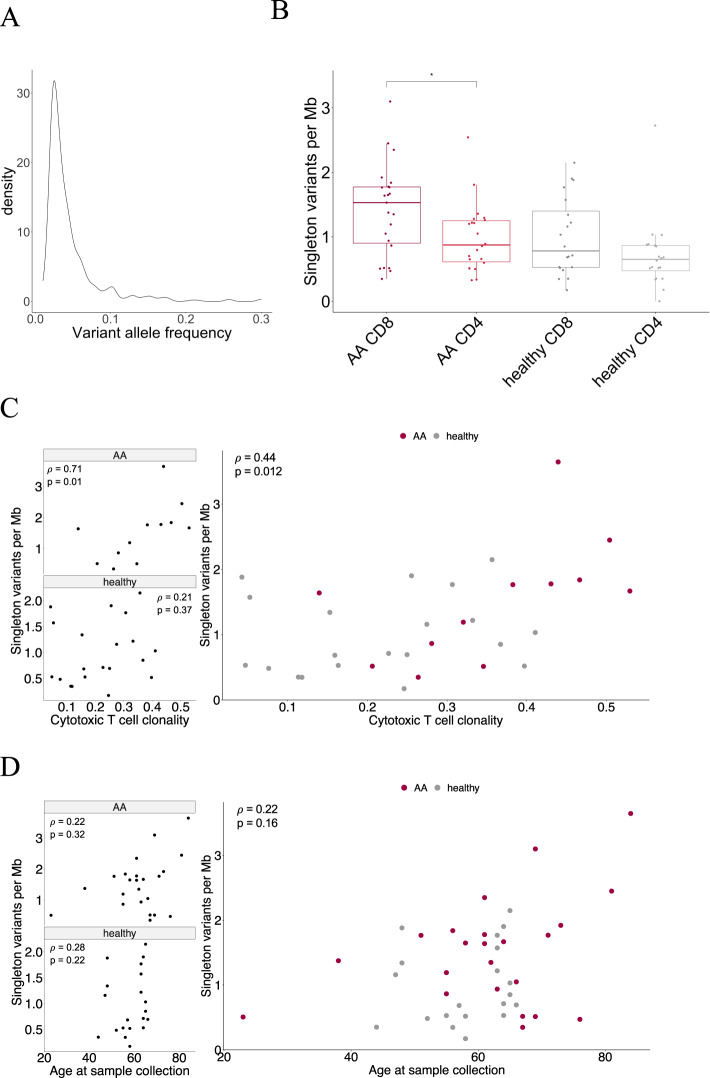
Variant allele frequency and adjusted somatic mutation burden. **A** Density plot of variant allele frequencies of CD4+ and CD8+ specific variants detected with immunogene panel sequencing. **B** Fraction-specific somatic mutation burden in AA and healthy samples. Mann–Whitney *U* test was used in pairwise comparisons within each disease group and cell fraction, only statistically significant (*p* < 0.05) differences are marked. **C** Mutation burden in CD8+ T cells was associated with T-cell clonality (Spearman *ρ* = 0.44, *p* = 0.012). Each group is shown separately on the left panel (Spearman test values for AA: *ρ* = 0.71, *p* = 0.012; healthy: *ρ* = 0.21, *p* = 0.37). **D** Number of somatic mutations in CD8+ T cells did not have significant correlation with age at sample collection, although a positive trend was observed.

We performed immunogene panel deep sequencing of CD4+ and CD8+ T cells from 24 AA patients and 20 healthy controls (age median [range], respectively: 63 [23–84] and 61 [44–66], Fig. S6). Mean sequencing depth was 299x [249x for AA and 359x for healthy samples] (Fig. S7A, B). We divided the variants to three categories: CD4+ specific or CD8+ specific variants (detected only in CD4+ or CD8+ T cells) and LP variants (detected in both CD4+ and CD8+ T cells, see “Methods”). We detected altogether 731 somatic variants in 519 genes in patients and healthy, with mean [median] of 8.3 [7] variants per sample (1.46 [1.22] variants/Mb) and mean variant allele frequency (VAF) of 4.5% (Fig. 1A). Nonsynonymous variants were enriched in AA patients compared to healthy controls (frequencies of nonsynonymous variants from all variants were 74.8% and 68.8% respectively, *p* = 0.058). The VAFs of CD4+ and CD8+ specific variants were not significantly different in AA patients (Fig. S7C). The complete list of variants discovered by the immunogene panel sequencing is presented in Table S3, and all nonsynonymous variants including their VAFs in individual patients are shown in Fig. S8.

Adjusted somatic mutation burden (see  Supplementary Methods) was the highest in CD8+ T cells in patients with AA (Fig. 1B). Both in AA and healthy controls, CD4+ cells had a lower mutation burden than CD8+ T cells (median for each group after outlier removal: AA CD8 1.53, AA CD4 0.87, healthy CD8 0.78, healthy CD4 0.65, Fig. 1B). In pairwise comparisons, AA CD8+ T cells had a significantly higher mutation burden compared to AA CD4+ T cells (*p* = 0.029). The number of variants detected in each sample did not correlate with sequencing depth (*ρ* = −0.15, *p* = 0.27, Fig. S7B).

We validated a selected set of mutations with amplicon sequencing, as shown in Table S4. Of 15 variants detected with immunopanel sequencing, 14 were confirmed with amplicon sequencing in at least one sample, if several samples from same patient were tested.

The T-cell clonality index for each CD8+ T-cell sample (see “Methods”) was defined using TCRB sequencing. The adjusted somatic CD8+ mutation burden significantly correlated with CD8+ T-cell clonality (*ρ* = 0.44, *p* = 0.012) but not with patient age (*ρ* = 0.22, *p* = 0.16) (Fig. 1C, D), despite a positive trend was observed with age and fraction-specific mutation burden in both CD8+ and CD4+ T cells (Fig. 1D, Fig. S9). When examined separately, a strong correlation was observed between the mutation load and cytotoxic T-cell clonality in AA but not in healthy controls (AA CD8 *ρ* = 0.71, *p* = 0.012, Fig. 1C).

### Somatic mutations in T cells accumulate into key immune regulatory pathways

**Fig. 2:**
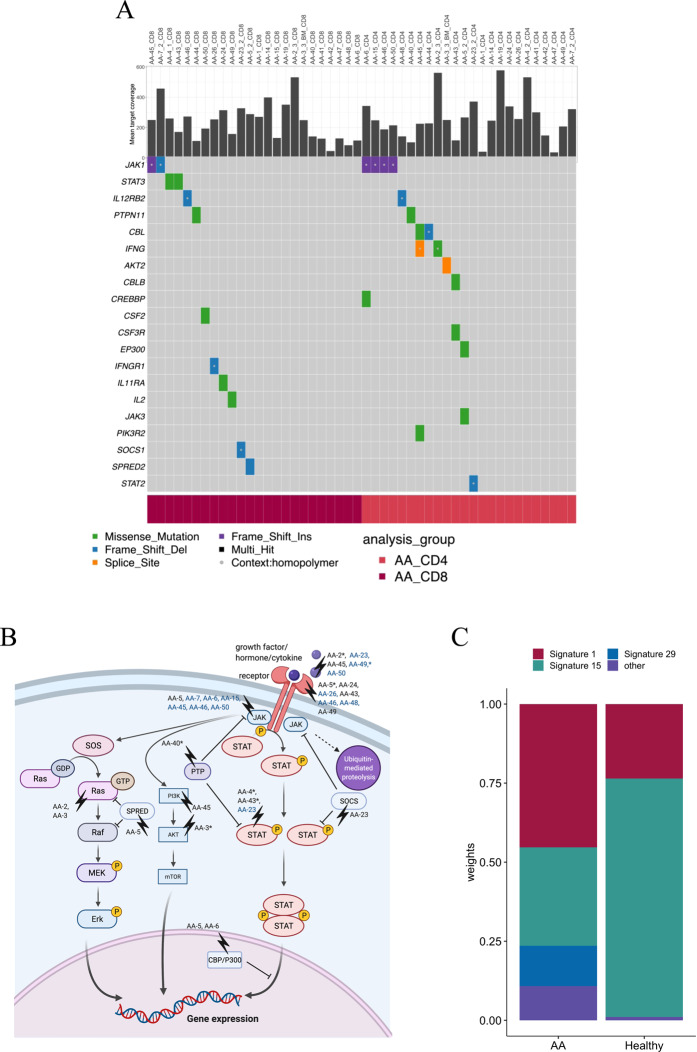
CD4+ or CD8+ specific somatic mutations in JAK-STAT pathway genes in AA patients. **A** Mean target coverage of each sample is presented in the top panel. Variants on homopolymer regions are marked with white dots. **B** Mutations marked on JAK-STAT signaling pathway based on KEGG database [38], complemented with closely related MAPK-Erk and PI3K-Akt-mTOR pathways. Patients with homopolymer region variants are colored with blue. Patients with pathogenic-predicted variants are marked with an asterisk (*). **C** Mutational signatures from pooled CD4+ and CD8+ T-cell somatic variants from each patient group.

CD8+ T cells in patients with AA harbored most potentially pathogenic variants affecting the JAK-STAT signaling (Fig. 2A, B) and MAPK signaling pathways (Fig. S10), annotated by Kyoto Encyclopedia of Genes and Genomes (KEGG) [38]. Mutations in these pathways were also detected in CD4+ T cells of AA patients.

JAK-STAT pathway mutations (in CD8+ or CD4+ T cells) were detected in 75% of AA patients (18/24) and 40% of healthy controls (8/20) (Fig. 2A, B, Fig. S11). The frequency of patients with CD4+ or CD8+ specific JAK-STAT pathway mutations was higher in AA than in healthy controls (*p* = 0.031). In CD8+ T cells, *JAK1* and *STAT3* were recurrently mutated (both 8.3%, 2/24 AA patients’ CD8+ T cells). *STAT3* mutations were only observed in AA patients’ CD8+ T cells. Both *STAT3* mutations were p.Y640F, which is a known mutation hotspot in *STAT3* exon 21 [27, 39]. *JAK1* mutations were in contrast detected in both CD4+ and CD8+ T cells and across patient and healthy groups. All *JAK1* mutations were located in homopolymer regions (Fig. 2A, homopolymer regions marked with white dots), known to be challenging in NGS based variant analysis.

The variant effects were predicted as described in Supplementary Methods. In the JAK-STAT pathway, genes with damaging-predicted mutations in immune-mediated AA patients included *IFNG, JAK3, PTPN11, STAT3, AKT2, IL2*, and *PTPN2* (Fig. S11). We discovered only one damaging-predicted CD4+ or CD8+ specific JAK-STAT pathway mutation per sample in AA patients.

Potentially pathogenic CD4+ or CD8+ specific MAPK signaling pathway variants were detected in 88% of AA patients (21/24). Also, 75% of healthy controls had CD4+ or CD8+ specific variants in MAPK signaling pathway. Recurrently mutated genes in immune-mediated AA patients included *ARRB2, CACNA1A, FLNB, NLK*, and *PPP3CC*. All nonsynonymous variants in MAPK pathway genes are presented in Fig. S10.

To investigate possible mechanisms inducing somatic mutations in T cells, we assessed mutational signature analysis of the variants. We included both synonymous and nonsynonymous single-nucleotide variants (SNVs) and pooled the variants across CD4+ and CD8+ T cells per group. Age-related signature 1 and defective mismatch-repair signature 15 accounted for the majority of mutations in both groups (Fig. 2C). Signature 29, which has been linked to guanine damage, was specifically detected in AA.

For SNV types across different groups, the most frequent alteration was C > T, a characteristic transversion type of replication as well as of aging-associated mutagenesis (Fig. S12A). Most nonsilent mutations were missense (Fig. S12B) and there was no significant difference in mutation types between groups (chi-squared test *p* value = 0.18).

### CH mutations transmit to T cells

**Fig. 3:**
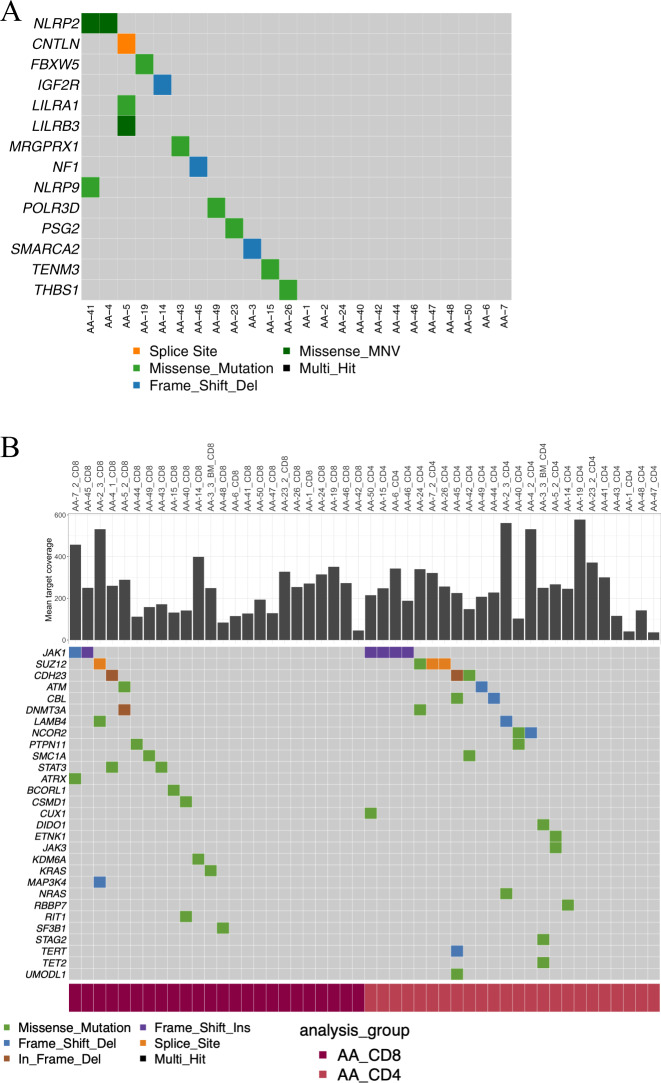
Somatic mutations related to lympho-myeloid clonal hematopoiesis in immunogene panel data. **A** All variants which were called from both CD4+ and CD8+ T cells in same patient and are hence interpreted to be derived from thymic precursors. MNV means two or more single-nucleotide variants in succession. **B** CD4+ and CD8+ specific variants in genes previously reported to be associated to CH in AA [13].

To understand timing of mutation acquisition, an integrative analysis of our panel and previously published exome sequencing data was performed. In our data, we found 15 nonsynonymous LP variants (occurring concurrently in both CD4+ and CD8+ T cells) in 14 AA patients (Fig. 3A and list of all variants in Table S3) with a mean allele frequency of 11.4% [median 7.8%]. Older AA patients inclined to have more LP variants (Fig. S13). With the exception of *NF1*, these mutated genes have not been previously linked to CH. While CD4+ and CD8+ T cells harbored many mutations in other genes linked to CH (e.g., *DNMT3A, BCORL1, TERT*, and *LAMB4*), they were not concurrently detected in both T-cell fractions (Fig. 3B).

By reanalyzing WES data from Yoshizato et al. [13], we investigated mutations in CH genes in T cells (Fig. S14). Most frequent mutations in CD3− cells (T-cell depleted fraction) were in *DNMT3A, BCOR/BCORL1*, and *ASXL1* genes (found in 7, 6, and 6 patients, respectively). In some cases, these mutations were also detected in CD3+ cells (T-cell enriched fraction) of the same patient, but in other instances they were present exclusively in CD3+ cells (*BCOR* and *BCORL1*). When detected in CD3+ cells, *ASXL1* or *DNMT3A* mutations were always concurrently present in CD3− cells, possibly due to a clonal advantage exclusive to myeloid cells. *JAK* mutations (*n* = 2) were found in both CD3− and CD3+ fractions. One *STAT*3 mutation (c.G1012T, p.V338F) was detected in CD3− MNC with 3.3% VAF. This particular mutation was not found in COSMIC v90 [39].

### Index patients

To understand the effect of the variants on cell phenotype, we performed paired single-cell RNA and TCRαβ sequencing (scRNA + TCRab-seq) analysis for samples from two index patients. We chose patients who carried mutations on JAK-STAT and MAPK signaling pathways and had available samples from different stages of disease.

**Fig. 4:**
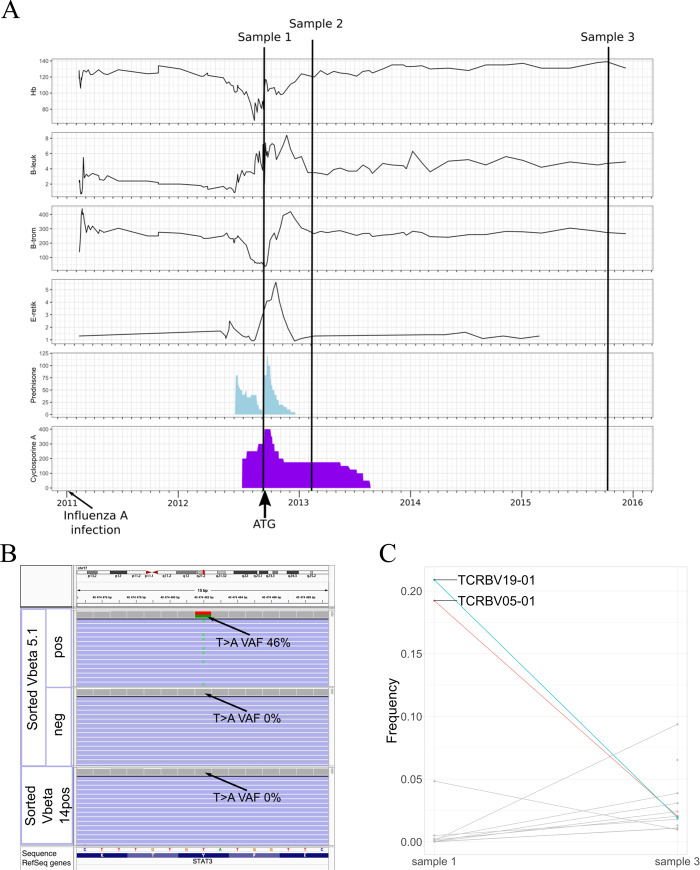
AA-4 clinical timeline and somatic mutations. **A** Samples analyzed with scRNA + TCRab-seq from index patient AA-4 are marked on the clinical timeline. In addition, BM biopsies were taken at the time of influenza A infection and preceding severe AA diagnosis. Blood counts are plotted in upper four panels (B-Hb = hemoglobin (g/l), B-neut = neutrophils (E9/l), B-trom = thrombocytes (E9/l), E-retik = reticulocyte percentage of erythrocytes). Corticosteroid (prednisolone) and cyclosporine A (CyA) dosages (mg) are plotted in two panels below. Timing of ATG treatment and influenza A infection are marked with arrows. **B** Somatic STAT3 Y640F mutation was validated with amplicon sequencing of sorted Vbeta 5.1+ fraction, but the mutation was neither detected in Vbeta 14+ nor in Vbeta 5.1− fraction (in sample 2). Picture of BAM files opened at variant site (chr17:42322464) in Integrative Genomics Viewer. **C** Most expanded CTL clones of AA-4 at diagnosis (sample 1) and remission (sample 3), based on TCRb sequencing. On the *x* axis are different time points and *y* axis shows the frequency of each TCRb clone from whole CTL TCRb repertoire. At diagnosis, there were two ~20% CTL clones, which significantly diminished during immunosuppressive treatment. Vbeta 5.1 and Vbeta 14 correspond to expanded T-cell clones TCRBV05-01 and TCRBV19-01, respectively.

Patient AA-4 was a 52-year-old previously healthy female who was first hospitalized due to severe H1N1-influenza infection, and afterward she gradually developed worsening pancytopenia. The first bone marrow sample was taken already at the time of H1N1 infection (because of neutropenia), but the actual diagnosis of very severe AA was made 17 months later (Fig. 4A). She was first treated with cyclosporine A (CsA) and corticosteroids (CS) without response. Two months later she was treated with equine antithymocyte globulin (ATG)-based immunosuppression which resulted in completely normalized blood counts after 4 months. Our first blood sample analyzed with scRNA + TCRab-seq was obtained immediately prior the ATG treatment and follow-up samples were received at 5 and 37 months. At the last sampling, the patient had been without any immunosuppressive treatment with normal blood counts for 25 months.

We found somatic *STAT3* mutation p.Y640F in CD8+ cells of AA-4, which was confirmed to be restricted to one CD8+ T-cell clone (Vbeta 5.1) by flow cytometry sorting and amplicon sequencing. The variant was not detected in Vbeta 5.1− cells or in the other expanded CD8+ T-cell clone (Vbeta 14+) (Fig. 4B). The VAF in sorted Vbeta 5.1 clone was 46%, indicating heterozygosity. The *STAT3* p.Y640F mutation was already detected with amplicon sequencing in the bone marrow sample taken at the time of influenza A infection (6% VAF), indicating infiltration of mutated T cells in the bone marrow at the first appearance of cytopenias. The VAF of the mutation in CD8+ T cells decreased from 16 to 1% (analyzed by the immunogene panel) after successful immunosuppression, consistent with shrinkage of the expanded T-cell clone TCRVB05-01 (corresponding to Vbeta 5.1) with TCRb sequencing (Fig. 4C).

**Fig. 5:**
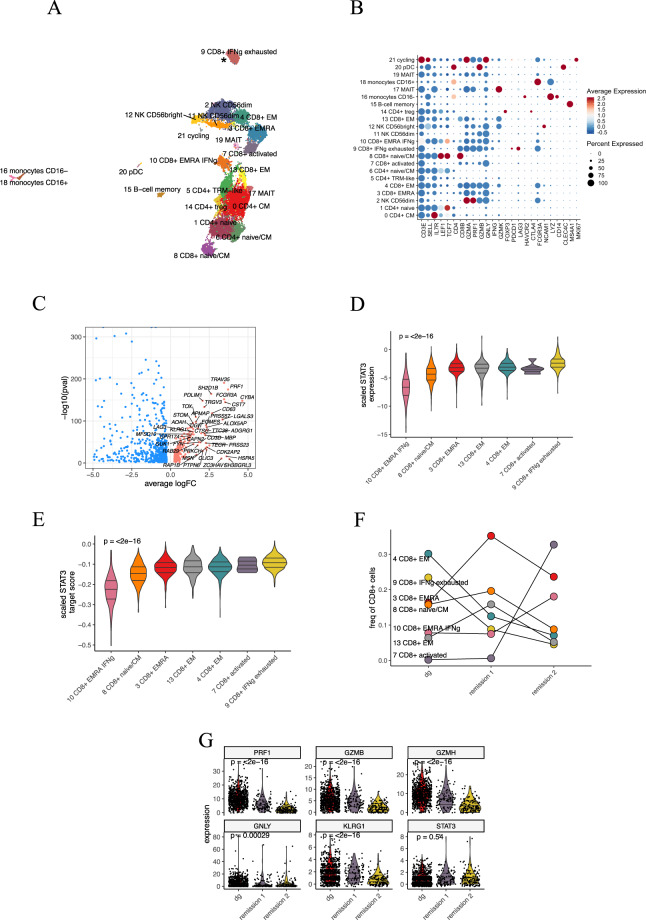
AA-4 scRNA + TCRab-seq analysis. **A** Two-dimensional UMAP projection of the transcriptomes of CD45+ lymphocyte cells pooled from three time points from peripheral blood. A total of 24,000 cells are annotated in 21 distinct clusters, 7 of which can be annotated as CD8+ T cells. *STAT3 mutated T-cell cluster. **B** Dot plot showing the canonical markers used to annotate the clusters. **C** Volcano plot showing differentially expressed genes between the TCRBV05-01 associated cluster (to the right, significant shown in red) against other CD8+ clusters (to the left, significant shown in blue). **D** Violin plot showing the scaled *STAT3* expression in CD8+ clusters at diagnosis. **E** Violin plot showing the score of genes having at least one occurence of the transcription factor binding site within 4 kb of STAT3 binding site at diagnosis. **F** The relative abundances of CD8+ clusters during treatment. **G** Violin plot showing the significantly downregulated cytotoxic genes (*PRF1*, *GZMB*, *GZMH*, *GNLY*, *LYZ*) and stable *STAT3* expression in the TCRBV05-01 associated cluster.

scRNA + TCRab-seq identified 21 different transcriptomic clusters including seven CD8+ T-cell clusters and five CD4+ T clusters (Fig. 5A, B). Almost all cells from *STAT3* mutated TCRBV05-01 clone (cluster 9) clustered separately. We compared the gene expression profile of this cluster to other CD8+ clusters and found cells to express aberrantly cytotoxic genes (such as *PRF1, NKG7, DUSP1*), genes associated with T-cell exhaustion (*LAG3, TIGIT*), and cytokines included in cell migration (*CCL3, CCL4, CCL3L1*, and *CCL4L1*, Fig. 5C).

At diagnosis, the TCRBV05-01 associated cluster expressed the highest level of *STAT3* and genes located within 4 kb of its binding site (Fig. 5D, E). After treatment, the TCRBV05-01 cluster diminished, concordant with the decreased *STAT3* VAF (Fig. 5F). The therapy also affected the phenotype of the cluster, as cells lost expression of multiple cytotoxic genes, including *PRF1, GZMB*, and *GNLY*. *STAT3* expression was not significantly altered between time points (Fig. 5G).

**Fig. 6:**
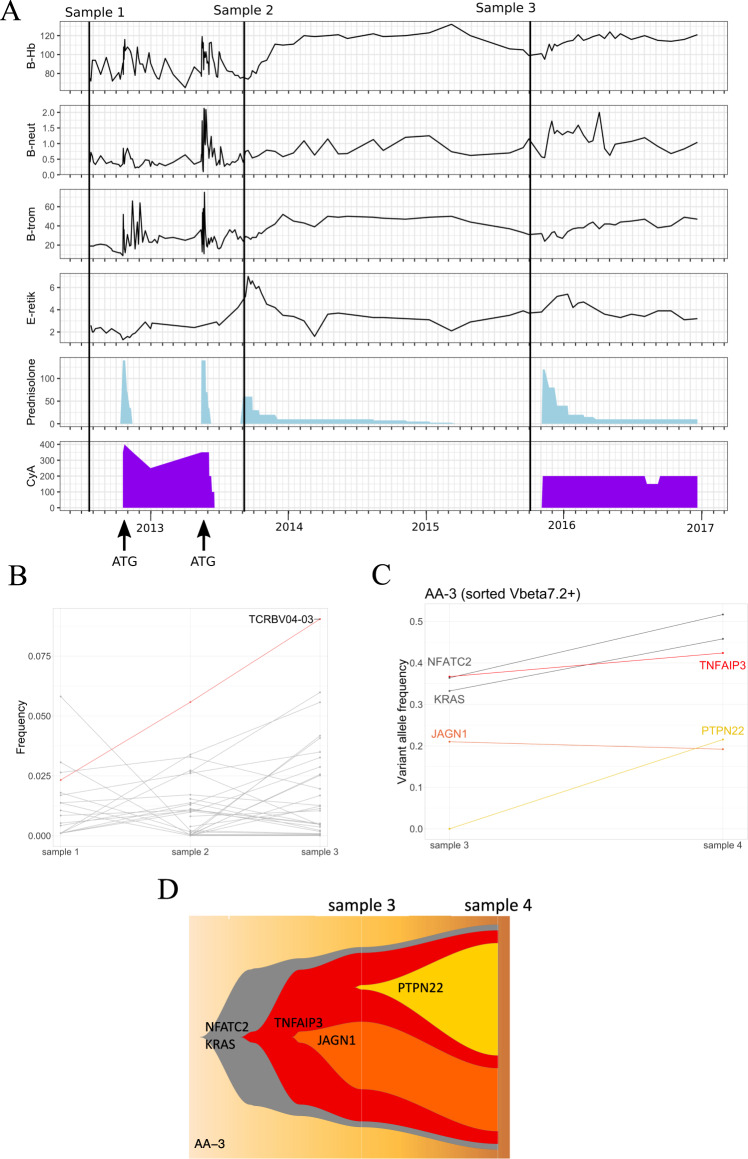
AA-3 clinical timeline and somatic mutations. **A** Samples analyzed with scRNA + TCRab-seq are marked on the clinical timeline. Blood counts are plotted in upper four panels (B-Hb = hemoglobin (g/l), B-neut = neutrophils (E9/l), B-trom = thrombocytes (E9/l), E-retik = reticulocyte percentage of erythrocytes). Corticosteroid (prednisolone) and cyclosporine A (CyA) dosages (mg) are plotted in two panels below. Timing of ATG treatments is marked with arrows. **B** In AA-3, TCRBV04-03 clone was expanding from sample 1 (diagnosis) to relapse (sample 3). On the *x* axis are different time points and *y* axis shows the frequency of each TCRb clone from whole CTL TCRb repertoire. **C** Amplicon sequencing confirmed all mutations in Vbeta 7.2+ (corresponding to TCRBV04-03) fraction of CD8+ T cells. *y* axis shows variant allele frequencies within Vbeta 7.2+ CD8+ T cells at different time points (shown at *x* axis). **D** Possible subclonal architecture of the mutations.

Patient AA-3 was a 58-year-old previously healthy female. She was diagnosed with severe AA after 2 years of macrocytosis and mild thrombocytopenia. Initial treatment included CsA and CS with no response, and 3 months after the diagnosis she was treated with equine ATG-based immunosuppression (Fig. 6A). No response was observed by 6 months, and rabbit ATG-based immunosuppressive therapy was administered. A year later (in summer 2014) hemoglobin was normal, and platelets and neutrophils temporarily improved. When the blood counts declined during the second year, the second remission was achieved with the reinitiation of CsA and CS; since then, she has continued on low-dose immunosuppression with moderate thrombocytopenia. The first sample was obtained before first ATG, the second sample after second ATG (before treatment response), and the third before the retreatment at the relapse.

Patient AA-3 had somatic mutations in several genes, including *KRAS, NFATC2, PTPN22, TNFAIP3*, and *JAGN1*. *KRAS* A146P, *JAGN1* T473A, and *TNFAIP3* D212fs mutations were predicted to be pathogenic, and same *KRAS* mutation has been found in several hematologic neoplasms (genomic mutation ID in COSMIC v91: COSV55541748). Similarly to AA-4, these mutations were confirmed to be restricted to a single CD8+ T-cell clone (Vbeta 7.2), as the variants were not detected in sorted monocytes, B cells, or Vbeta 7.2− T cells. Vbeta 7.2 clone, corresponding to TCRBV04-03 in TCRb deep sequencing, increased despite immunosuppressive treatment (Fig. 6B). VAFs in sorted Vbeta 7.2 clone from sample 3 and additional sample taken a year later are shown in Fig. 6C. An additional fourth sample was only used for validation of variants with amplicon sequencing, which revealed a possible subclonal architecture, as presented in Fig. 6D.

**Fig. 7:**
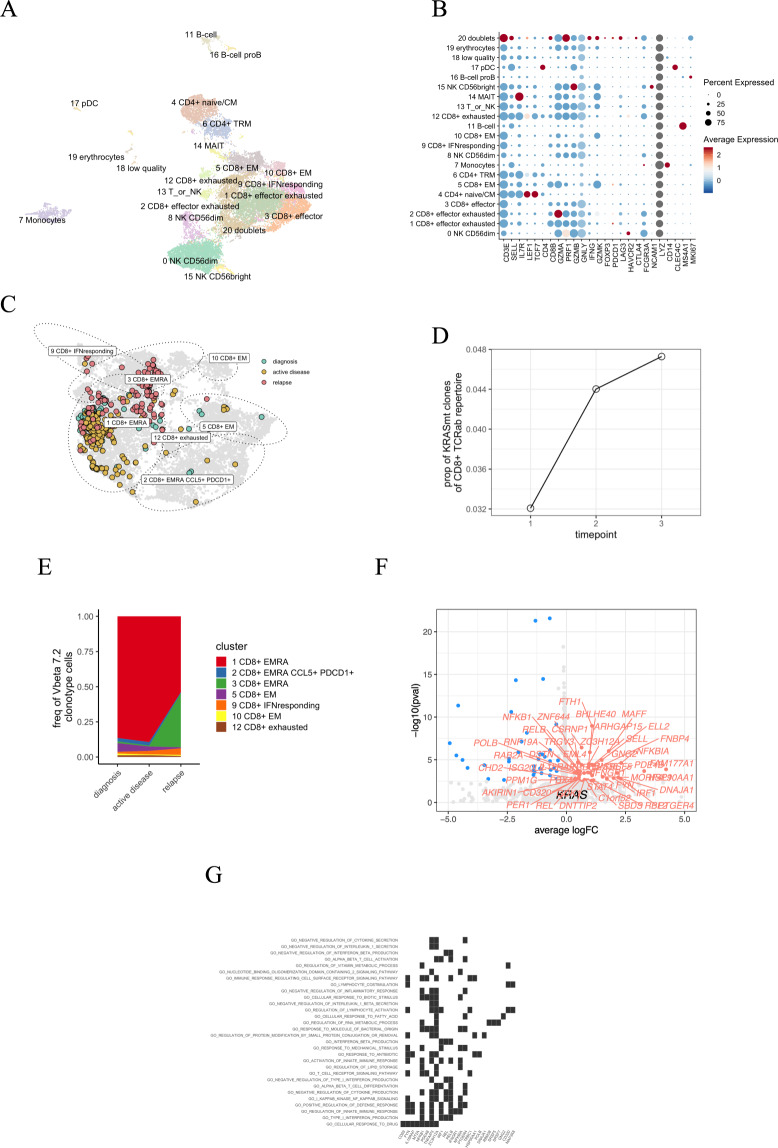
AA-3 scRNA + TCRab-seq analysis. **A** Two-dimensional UMAP projection of the transcriptomes of CD45+ lymphocyte cells pooled from three time points from bone marrow. A total of 22,407 cells are annotated in 21 distinct clusters, 7 of which that can be annotated as CD8+ T cells but only 2 as CD4+ T cells. **B** Dot plot showing the canonical markers used to annotate the clusters. **C** Focused two-dimensional UMAP projection of re-embedded CD8+ lymphocyte cells. The encircled dots are the TCRBV04-03 clone colored by different time points, where turquoise is the diagnostic (sample 1), yellow is follow-up (sample 2), and red is from relapse (sample 3). Most of the TCRBV04-03 clonotype cells can be classified as 1 CD8+ T_EMRA_. **D** The proportion of TCRBV04-03 clonotype cells from the total CD8+ repertoire from the single-cell TCRab data. **E** The relative abundances of TCRBV04-03 clone’s phenotype during treatment. **F** Volcano plot showing differentially expressed genes between clonotype of interest (to the right, significant shown in red) and cells from other clonotypes from the 1 CD8+ T_EMRA_ cluster (to the left, significant shown in blue). **G** Gene set enrichment analysis (GSEA) results from the differential expression analysis. Shown here are GO categories enriched (FDR qval < 0.05) to the TCRBV04-03 clone.

In single-cell analysis of this patient’s CD45+ lymphocyte cells from the bone marrow samples at three time points, we identified 21 different clusters including seven CD8+ T-cell clusters but only two CD4+ T clusters (Fig. 7A, B). We identified 350 cells with matching TCRb nucleotide sequence to the TCRBV04-03. Most of the cells from the clone were CD8+ terminally differentiated effector memory cells (T_EMRA_), supporting the cytotoxic role of the TCRBV04-03 clone (Fig. 7C).

The TCRBV04-03 clone increased after the CsA, CS, and repeated ATG administration, and it was largest in the relapse sample (4.8% of the whole CD8+ repertoire, Fig. 7D). Similarly, the phenotype of the TCRBV04-03 clone changed from diagnosis to relapse (Fig. 7C, E).

To understand the effect of the somatic mutations on the phenotype of the TCRBV04-03 clonotype, we performed differential expression analysis between the T_EMRA_ cells, comparing the cells from the TCRBV04-03 clonotype against the other cells in T_EMRA_ cluster. In the relapse sample, the number of differentially expressed genes between the TCRBV04-03 clonotype in comparison to other T_EMRA_ cells was the highest compared to other samples (56 genes up in TCRBV04-03 clonotype and 77 genes up in other T_EMRA_ cells, genes listed in Table S7). Several genes associated with the immune response such as *STAT4, NFKB1*, and *IFNGR1* were upregulated in the TCRBV04-03 clonotype (Fig. 7F). In gene set enrichment analysis, the upregulated pathways included type I interferon production, NF-kappaB signaling, and lymphocyte activation (Fig. 7G).

## Discussion

Accumulation of somatic mutations is inherent in normal cell division and aging. Mutations can lead to abnormal cell function and uncontrolled cell proliferation, as is well established in cancer. Recently, it has also been shown that somatic mutations occur in healthy human tissues [40–43] and can play a role in the pathogenesis of other nonmalignant diseases [44]. In the current work, we addressed the key questions of somatic mutations in T cells in patients with immune-mediated AA: in which cell subset they occur, at which state of immune cell development they emerge, which genes and pathways are affected, and correlation with clinical findings.

By gene panel sequencing of T cells, we discovered that somatic variants in immune-related genes are strikingly common in T cells. The mutation burden in CD8+ T cells was higher than in CD4+ T cells. This observation is in line with previous findings [21, 22, 24] and has both technical and biological explanations. CD8+ T cells expand to large clones [45], especially following strong antigen stimuli such as EBV and CMV infections [46–48]. During rapid proliferation cells are more susceptible to mutations. Larger T-cell clones have higher VAFs, which facilitates mutation detection. However, in our data, the mutation burden correlated with the T-cell clonality within AA patients’ CD8+ T cells but not in healthy CD8+ T cells. This observation implies that a technical detection threshold could not alone explain higher mutation burden in CD8+ T cells of AA patients.

In accordance with our previous findings [21, 22], we were able to confirm that CD8+ specific somatic mutations were restricted to single T-cell clones, indicating post-thymic emergence. We were not able to study the clonal architecture of somatic variants in the whole cohort, as the majority of the variants had low VAFs and may have occurred in different T-cell clones. Thus, the hierarchical ordering of variants based on VAFs does not necessarily reflect the true order of mutation appearance, as other factors, such as antigen-driven expansion of T cells, may distort the hierarchy. The clonal structure of mutations have been recently studied in myeloid malignancies with single-cell DNA sequencing [49] and that approach could elucidate the role and order of different mutations in T cells as well.

We also showed that mutations in genes related to CH are common in T cells, and CH mutation transmission to T cells was frequently observed in the exome sequencing dataset of AA patients’ CD3+ and CD3− MNCs [13]. The lack of sequencing data from myeloid compartment distinctly limits our analysis. Previously, it has been shown that CH mutations are more rare in T cells compared to myeloid cells [50–52], but as the majority of publications on CH are based on whole blood or total MNC fraction sequencing, the mutation-specific patterns and prevalence of CH-related mutations in T cells remain to be conclusively defined.

In immune-mediated AA, we found that T-cell somatic mutations were especially frequent in the JAK-STAT and MAPK pathways. It should be noted that healthy controls also harbored mutations in both pathways, which may implicate that mutations in these pathways may be beneficial to T cells in general. Both of these pathways are important for T-cell development and function. The JAK-STAT pathway plays a central role in cytokine signaling transmitting activation and proliferation signals in T cells [53]. MAPK pathway is a major pathway induced by TCR stimulation [54] and it also regulates apoptosis [55]. However, with our panel, we were only able to analyze mutations in 2533 preselected genes, and the full spectrum of somatic mutations in T cells needs to be studied in future with exome or whole genome sequencing.

To address the effect of somatic mutations on cell phenotype, we combined single-cell RNA sequencing with TCRab sequencing. *STAT3* p.Y640F mutated CD8+ T cells had a strikingly altered immunophenotype compared to other CD8+ T cells, forming their own unique phenotype cluster. The clone also showed higher expression of *STAT3* target genes, which is consistent with previous studies showing *STAT3* activation following p.Y640F mutation in LGL leukemia, anaplastic large cell lymphoma, inflammatory hepatocellular adenomas, and murine hematopoietic stem cells [27, 56–58]. *STAT3* has been shown to mediate effector T-cell resistance to suppression in patients with type 1 diabetes [59].

*STAT3* mutated cells had highly cytotoxic immune phenotype similarly as *KRAS* mutated T cells in the other AA index patient. The treatment response in these two patients was different and correlated well with findings in the clone size and phenotype. Thus, our results suggest that immunosuppressive treatment may alter not only the clone size but also the cytotoxic function of mutated CD8+ T cells, and that these changes may be linked to clinical outcomes. To generalize our results and to study effect of different mutations on clinical outcomes, a bigger cohort of AA patients would be needed with detailed clinical information available.

As the antigen target in immune-mediated AA is still unknown, we were not able to test the autoreactivity of clonal T cells. It is also undetermined whether the somatic mutations are initial drivers or secondary events following autoantigen-driven clonal expansions [60]. However, as both in our study and previous studies [21, 26] it has been shown that mutated T-cell clones persist for several years, they may maintain the aberrant immune responses. Growing evidence suggests that in myeloid cells, somatic mutations are associated with inflammatory phenotypes and increased risk for cardiovascular diseases [61–63]. Besides, donor CH may be related to enhanced adaptive immune response in bone marrow transplant recipients, as recipients with CH have lower relapse rate but higher rates for graft-versus-host disease [64]. Furthermore, Fraietta et al. showed that a TET2-deficient CAR-T cell clone had less T-cell exhaustion in response to stimulus and killed tumor cells more efficiently [65].

In conclusion, somatic mutations in T cells particularly in the JAK-STAT and MAPK pathways are common in patients with immune-mediated AA. Mutations are associated with T-cell clonality but not distinctly with age, differentiating them from typical CH mutations. Our findings imply that somatic mutations are one putative mechanism of aberrant T-cell activation. Treatments that target the mutated clones could have therapeutic value in immune-mediated AA.

## Supplementary information

Supplemental Material

Supplemental table S1

Supplemental table S2

Supplemental table S3

Supplemental table S4

Supplemental table S5

Supplemental table S6

## Data Availability

The scRNA + TCRab-seq raw data have been deposited at the European Genome-phenome Archive (https://ega-archive.org), which are hosted by the EBI and the CRG, under accession number EGAS00001004994. The processed scRNA + TCRab-seq data are available in ArrayExpress database at EMBL-EBI (www.ebi.ac.uk/arrayexpress) under accession number E-MTAB-9969. Immunogene panel sequencing raw data are available from the corresponding author upon request.

## Code availability

The source code of single-cell sequencing bioinformatic analyses is available at GitHub (https://github.com/janihuuh/aa_som_mut_manu).
