# Supplementary material for: Somatic mutations in lymphocytes in patients with immune-mediated aplastic anemia
Source: Leukemia. 2021 Mar 30;35(5):1365–79. doi: 10.1038/s41375-021-01231-3 (PMC8102188; doi:10.1038/s41375-021-01231-3)
Supplement: Supplementary file 1 — Supplemental Material [file 41375_2021_1231_MOESM1_ESM.pdf]

Supplementary Methods

Sample preparation and immunogene panel sequencing

Mononuclear cells (MNC) were enriched from peripheral blood or bone marrow samples with Ficoll density gradient centrifugation (Ficoll-Paque PLUS, GE Healthcare). CD8+ and CD4+ T cell fractions were separated via positive selection by immunomagnetic bead sorting (Miltenyi Biotec) according to manufacturer's instructions or by flow cytometry (FACSAria II, Beckton Dickinson). Purity of CD4+ and CD8+ T cells was analyzed by flow cytometry sorting and purities were 92-99% of CD3+ cells. Purity analysis and sorting was performed with following antibody mixture (Cat. No. 342417) from Beckton Dickinson (BD): anti-CD45 (2D1, PerCP), anti-CD3 (SK7, FITC), anti-CD4 (SK3, APC), and anti-CD8 (SK1, PE-Cy7). DNA from separated CD4+ and CD8+ T cells was isolated with NucleoSpin Tissue kit (Macherey-Nagel, Cat. No. 740952) according to manufacturer's instructions. Library preparation, target genome capture and sequencing was performed as described previously (1).

Identification of potentially pathogenic somatic variants

Variant identification was performed with Genome Analysis Toolkit (GATK) and involved an initial variant discovery on individual samples and a subsequent genotyping of all variants across all samples. Briefly, pre-processing of sequencing data, read mapping to GRCh38 reference, and discovery of short somatic variants followed our previous procedure (2,3). Variants were discovered in tumor-only mode without a paired normal as well as by pairing samples with their matched counterparts (*i.e.* CD4+ datasets paired with CD8+ datasets and vice versa and CD3+ datasets paired with CD3neg datasets and vice versa). MuTect2 was used to make variant calls. Recurrent variant calling artifacts were in exome sequencing analyses filtered using a panel of normals (PON) created from the exome data of 24 healthy unrelated Finnish individuals and in panel sequencing analyses using a panel of normals created from immunogene panel healthy T cell samples. For the genotyping analysis, variant calls passing filters and supported by ten or more reads were aggregated across samples and supplemented by a set known *STAT3* lost-of-function variants[4] and hotspot variants (detected ≥30 samples and constituting ≥1% of the listed mutations of the each gene in COSMIC v90 (4)) in genes recurrently mutated in AA (5) or T cell neoplasms (6) (Supplementary table S2). These variants were then genotyped across samples in tumor-only mode using GATK-4.1.3.0 Mutect2 program. Finally, variant calls obtained from genotyping analyses

were filtered for vector contamination, RNA or pseudogene artefacts as described previously (3) to distinguish variants with a low variant allele frequency from technical or biological artefacts.

Variant annotation was performed with Annovar tool15 against the RefGene database and variant filtering using in-house solution. At first, variant calls were normalized using bcftools16. Variants other than those passing all MuTect2 filters and located in intronic and intergenic regions were then filtered as well as variants with a total depth ≤30, a strand-specific depth <1 in both directions, <1 read in the F1R2 configuration, <1 read in the F2R1 configuration, quality value ≤40, variant allele frequency ≤2% or ≥30%, strand odd ratio for SNVs ≥3.00, strand odd ratio for indels ≥11.00, minor allele frequency ≥1% in the 1KG database and EPS database, minor allele frequency ≥0.1% in general, American, African, Finnish, East Asian, and Non-Finnish European ExAC, gnomAD v2 exome, or gnomAD v2 genome databases, and minor allele frequency ≥0.1% in male, female, or gender unspecific general, Amish, American, African, Ashkenazi Jewish, other, Finnish, South Asian, East Asian, and Non-Finnish European gnomAD v3 databases. Variants with a variant allele frequency 1-2% were accepted, if supported by five or more COSMIC17 samples. Finally, variants were filtered against variants detected in two or more immunogene panel healthy T cell samples and skin WES samples. For functional analyses, the variant call set was further filtered by removing synonymous mutations and non-frameshift variants. Potentially pathogenic variants that were identified in both CD4+ and CD8+ T datasets of same patient were interpreted as lymphoid precursor (LP) variants. Rest of the variants were categorized as CD8+ or CD4+ specific variants. Sequencing coverage was computed using Samtools (7) depth and by restricting coverage computation to panel regions and/or known RefSeq exons.

Somatic mutation burden analysis

As variants seen recurrently in healthy T cell samples were used to filter variants, an adjusted somatic mutation burden was computed for each sample by taking into account only singleton variants passing the filtering process. Adjusted somatic mutation burden was calculated separately for LP variants and fraction-specific variants in CD4+ and CD8+ T cells of each patient. Total number of variants was divided by the number of exonic bases with a depth ≥30 per sample. In group comparisons, outliers were excluded by removing samples whose adjusted mutation burden was more than 1.5 fold above 3rd or below 1st quartile.

Variant effect predictions

Variant effects were predicted with eight approaches: SIFT (8), Polyphen-2 (HVar and HDiv) (9), likelihood ratio test (10), MutationTaster (11), MutationAssessor (12), FATHMM (13), VEST3 (14,15) and CADD (16,17). Conservation scores were predicted using three approaches: SiPhy (18) and phyloP (placental and vertebrate) (19). For conservation scores, we predicted variants with score of >1.6 for PhyloP and >12.17 for SiPhy to be pathogenic, as suggested by Dong et al (20). Variant effect and conservation results were aggregated using a majority rule (*i.e.* the variant was deemed as damaging if more than 50% of predictions categorized it as "damaging", "pathogenic", "possibly pathogenic", "medium" or "high").

Mutational signature analysis

All synonymous and non-synonymous variants passing the filtering process were used in mutational signature analysis. Identification of mutational signatures was done using the deconstructSigs18 software with default parameters and using cancer profiles downloaded from the COSMIC web site on September 2017. Function mapSeqlevels from the package GenomeInfoDb was used to convert EnsEMBL chromosome nomenclature to UCSC nomenclature.

Amplicon validation

Mutations found in the immunopanel sequencing (Supplementary table S4) were validated by amplicon sequencing as previously described (21) with small modifications. 2-step PCR protocol was used and sample pools were sequenced with Illumina HiSeq System using Illumina HiSeq Reagent Kit v4 100 cycles kit or Illumina MiSeq System using MiSeq 600 cycles kit (Illumina, San Diego, CA, USA).

Amplicon read alignment was performed with Bowtie2. GATK IndelRealigner was used for local realignment near indels. A variant was called, if variant count was >5 and base frequency was 0.5% of all reads covering a given a position. Variants with the base quality frequency ratio (ratio of number of variant calls/numbers of all bases and quality sum of variant calls/quality sum of all bases at the position) <0.9 were excluded. Somatic variant was considered to be true if it was called and passed all filters in both immunogene panel sequencing and amplicon sequencing with similar VAF.

TCR Vβ family based flow cytometry analysis and sorting

TCR Vβ families of CD4+ and CD8+ T cells were analyzed from frozen MNC by flow cytometry-based antibody staining using IOTest® Beta Mark TCR Vβ Repertoire Kit recognizing 24 members of TCR β chain, which covers about 70% of the normal human TCR Vβ repertoire (cat. no: IM3497, Beckman Coulter). MNC samples were stained after thawing with anti-CD3 (SK7, BD, cat. no 345767), anti-CD4 (SK3, BD, cat. no 345770), and anti-CD8 (SK-1, BD, cat. no 335822) and the panel of TCR Vβ antibodies. All antibodies were used according to the manufacturers' instructions. Stained cells were further analysed and sorted using FACSAria II (BD).

Single-cell gene expression and V(D)J transcript profiling

Frozen MNC from PB or BM were sorted with BD Influx Cell sorter and the gene and V(D)J transcript profiles were studied with 10x Genomics Chromium Single Cell V(D)J and 5' Gene Expression platform. When thawing, the cryo-preserved samples were resuspended to 13ml of +37°C plain RPMI (Roswell Park Memorial Institute) and after centrifugation of 5 minutes at 300g, washed with PBS + 2mM EDTA buffer. After 2nd 5 minute centrifugation at 300g, cells were resuspended to PBS + 0.05% BSA in concentration of 10,000 cells/ul and 2ul of CD45-APC-H7 (2D1, BD, cat. no 641417) and 5ul CD34 (8G12, BD, cat.no 345801) antibodies per 1 million cells were added. Samples were incubated with the antibodies for 15 minutes in room temperature. Cells were washed with 2ml of PBS-BSA buffer (300g/5min), resuspended to RPMI and kept on ice before and after sorting. Sorting was performed with BD Influx Cell sorter with gating strategy as presented in Figure S2. 300,000 target cells were collected to Protein Lo-Bind tubes (Eppendorf, cat. no 0030108). After sorting, single-cell samples were partitioned using a Chromium Controller (10X Genomics) and scRNA-seq and TCRαβ-libraries were prepared using Chromium Single Cell 5' Library & Gel Bead Kit (10X Genomics), according to manufacturer's instructions (CG000086 Rev D). 12,000 cells from each sample, suspended in 0.04% BSA in PBS, were loaded on the Chromium Single Cell A Chip. During the run, single-cell barcoded cDNA is generated in nanodroplet partitions. The droplets were subsequently reversed and the remaining steps were performed in bulk. Full length cDNA was amplified using 14 cycles of PCR (Veriti, Applied Biosystems). TCR cDNA was further amplified in a hemi-nested PCR reaction using Chromium Single Cell Human T Cell V(D)J Enrichment Kit (10X Genomics). Finally, the total cDNA and the TCR-enriched cDNA were subjected to fragmentation, end repair and A-tailing, adaptor ligation, and sample index PCR (14 and 9 cycles, respectively). All libraries were sequenced using NovaSeq 6000 system (Illumina), S1 flowcell with the following read length configuration for gene

expression libraries: Read1=26, i7=8, i5=0, Read2=91. Length configurations used for TCR-enriched libraries: Read1=150, i7=8, i5=0, Read2=150. The raw data was processed using Cell Ranger 3.0.1 pipelines. 10X Genomix pipelines “cellranger mkfastq” was used to produce FASTQ (raw data) files, “cellranger count” to perform alignment, filtering and UMI counting for the 5' gene expression data and "cellranger vdj" to perform V(D)J sequence assembly and paired clonotype calling for the V(D)J data.

Single-cell RNA-sequencing data analysis

Cells were subject to quality control. Cells with a high amount of mitochondrial transcripts (>15% of all UMI counts) or ribosomal transcripts (>50%), cells with less than 100 genes or over 4500 genes expressed, cells expressing a low or high (<25% or >60%) amount of house-keeping genes, or cells with a low or high read depth (<500 or >30 000) were excluded. Quality control measures are illustrated in Figure S3 for AA-4 and Figure S4 for AA-3. In total, 7 822 – 15 651 cells per sample were captured.

To overcome batch-effect, we used a recently described probabilistic framework to overcome different nuisance factors of variation in an unsupervised manner with deep generative modelling as described elsewhere (22). Briefly, the transcriptome of each cell is encoded through a nonlinear transformation into a low-dimensional, batch corrected latent embedding. The latent embedding was then used for graph-based clustering implemented in Seurat (3.0.0) and UMAP-dimensionality reduction with default parameters in RunUMAP function. TCR-related (V, D and J) genes were excluded from the clustering. The number of latent dimensions used were 16 (for AA-3) and 22 (for AA-4). Differential expression analyses were performed based on the t-test, as suggested by Soneson and Robinson (23), where Bonferroni adjusted p-values below 0.05 where denoted as significant. Clusters were annotated using differentially expressed genes, comparison to bulk-RNAseq based on sorted immune subsets and canonical markers (Figure S5). List of differentially expressed genes for each cluster are provided in Supplementary Table S5 for AA-4 and S6 for AA-3. The V(D)J sequences of each cell were integrated into the Seurat object as metadata for gene expression and clonotype analysis. Clonotypes were identified based on the total nucleotide level TCRα and TCRβ. Gene Set Enrichment Analysis (GSEA) (software.broadinstitute.org/gsea/index.jsp) between groups was based on ordered gene lists by fold-change. Overlap with GO and HALLMARK-categories was assessed and the False Discovery Rate (FDR) calculated while the number of permutations was 1000. The source code will be available at github (https://github.com/janihuuh).

Supplementary Figures and tables

Figure S1.

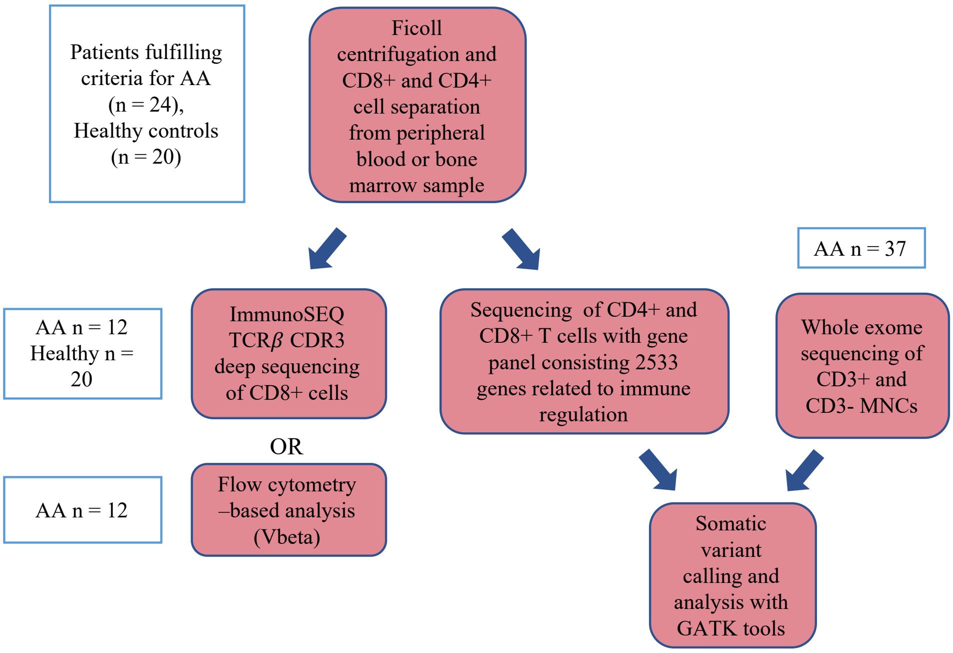

Study design. Bead separated CD8+ and CD4+ T cells were sequenced with immunogene panel to characterize somatic mutations in lymphocytes. GATK tools were used in the analysis. We also performed TCRb sequencing of all available patients' CD8+ T cells to understand the clonal dynamics of cytotoxic T cell repertoire. Whole exome sequencing data of CD3+ and CD3neg MNCs was incorporated into the variant analysis.

Figure S2.

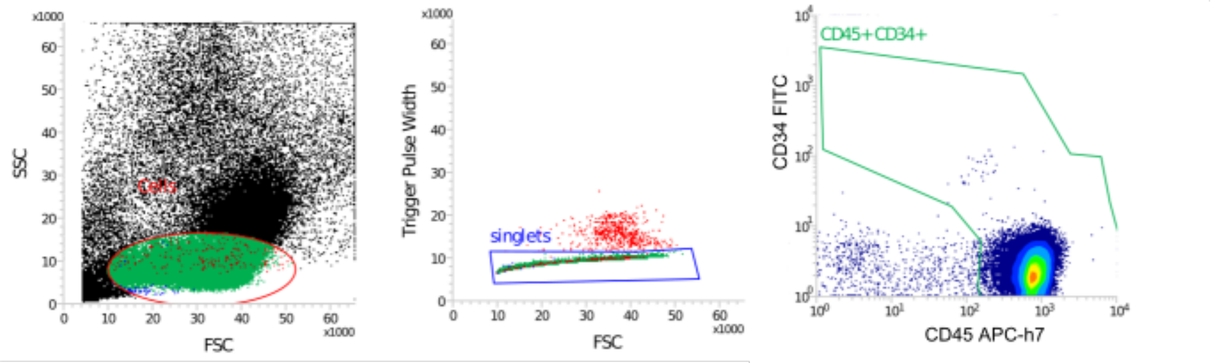

Sorting strategy for samples analyzed by scRNA+TCRab-seq. Samples were stained with anti-CD45 and anti-CD34 antibodies. With flow cytometry sorting, we selected the CD45+ and CD34+ cell population (circled with green in the most right panel) for analysis. SSC = side scatter, FSC = forward scatter.

Figure S3.

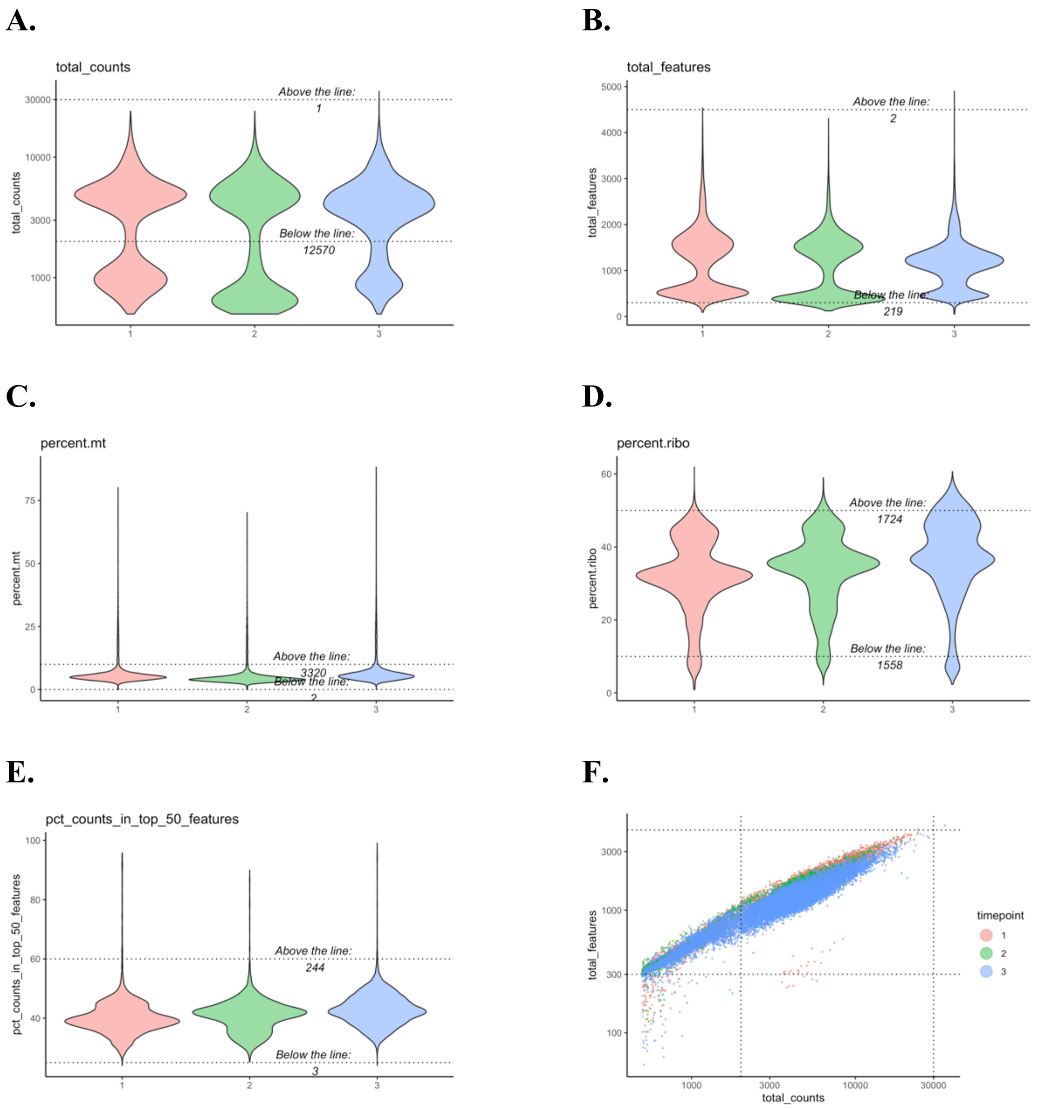

Quality control of scRNA+TCR-seq data on patient AA-4. Timepoints 1-3 are presented with same colours in all plots and treshold values used in filtering are marked with dashed line. Number of filtered cells is written next to treshold lines. (A) Number of reads per cell. (B) Number of expressed genes per cell. (B) Mitochondrial gene expression as a percent of all expressed genes per cell. (D) Percent of ribosomal genes of all expressed genes. (F) Percent of household genes of all expressed genes. (E) Number of expressed genes is shown on y axis and number of reads per cell are shown on x axis.

Figure S4.

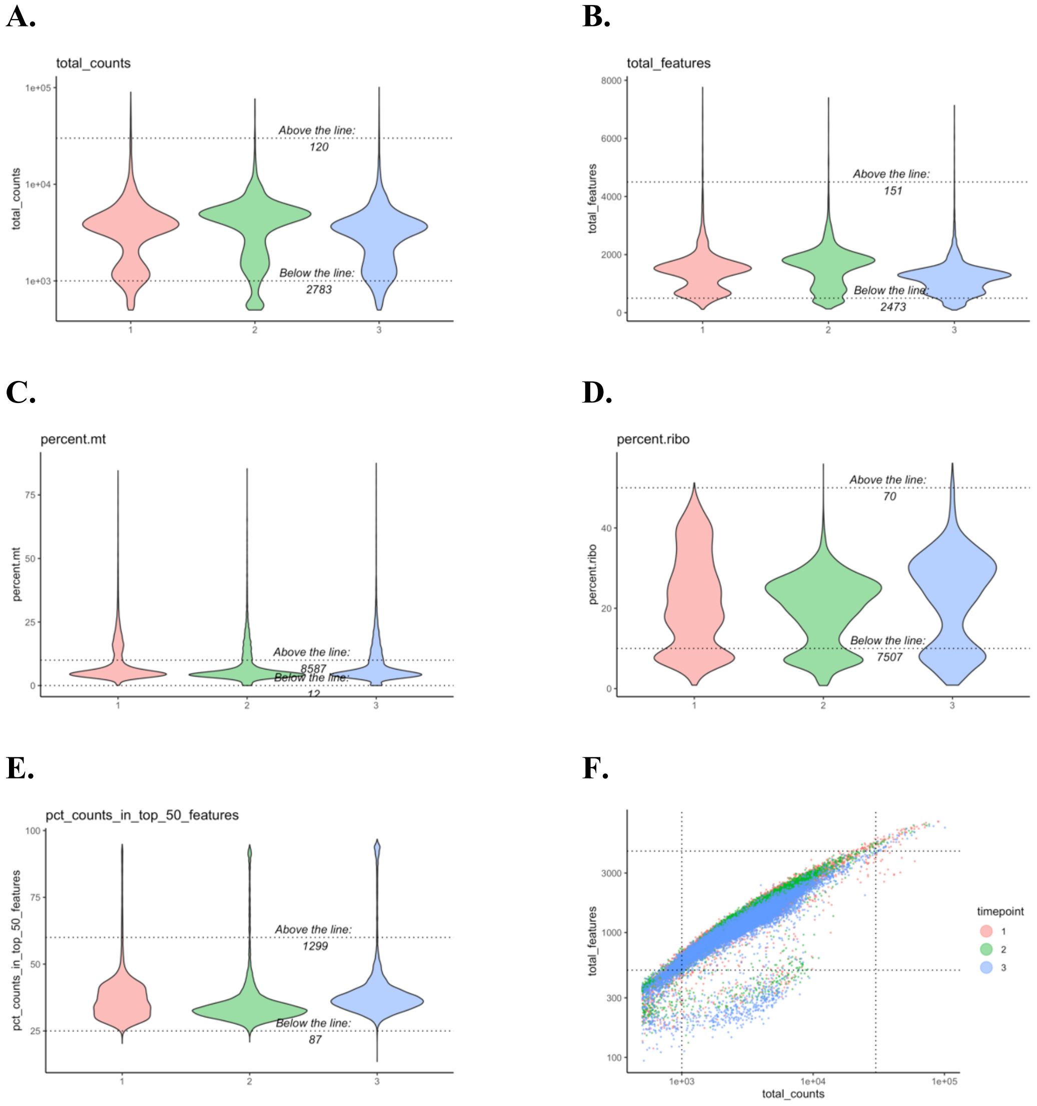

Quality control of scRNA+TCR-seq data on patient AA-3 (A) Number of reads per cell. (B) Number of expressed genes per cell. (B) Mitochondrial gene expression as a percent of all expressed genes per cell. (D) Percent of ribosomal genes of all expressed genes. (F) Percent of household genes of all expressed genes. (E) Number of expressed genes is shown on y axis and number of reads per cell are shown on x axis. Timepoints 1-3 are presented with same colours in all plots and treshold values used in filtering are marked with dashed line. Number of filtered cells is written next to treshold lines.

Figure S5.

A.

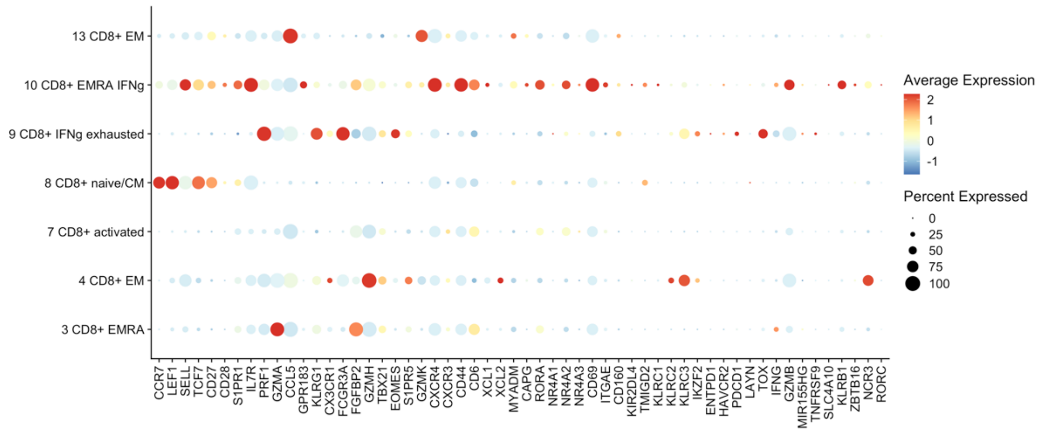

B.

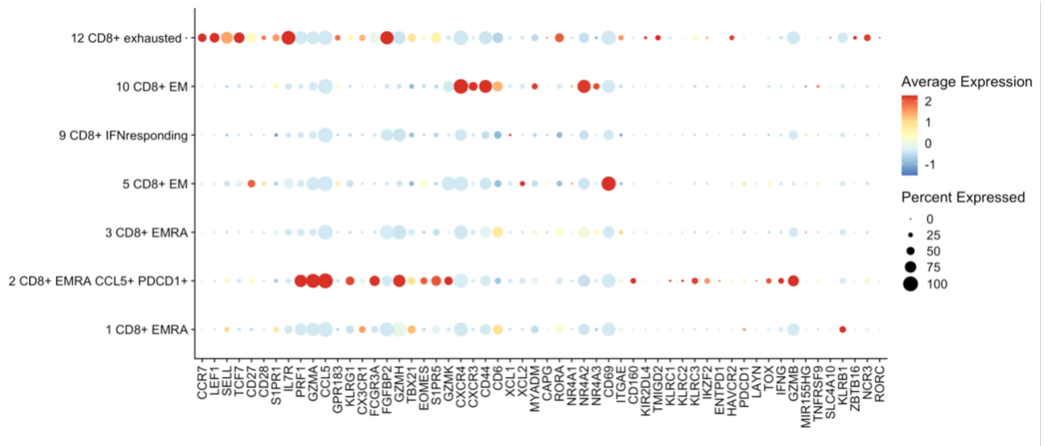

Cluster annotation. Shown markers are signature genes expressed by CD8+ T cells (27) of patient AA-4 (A) and AA-3 (B).

Figure S6.

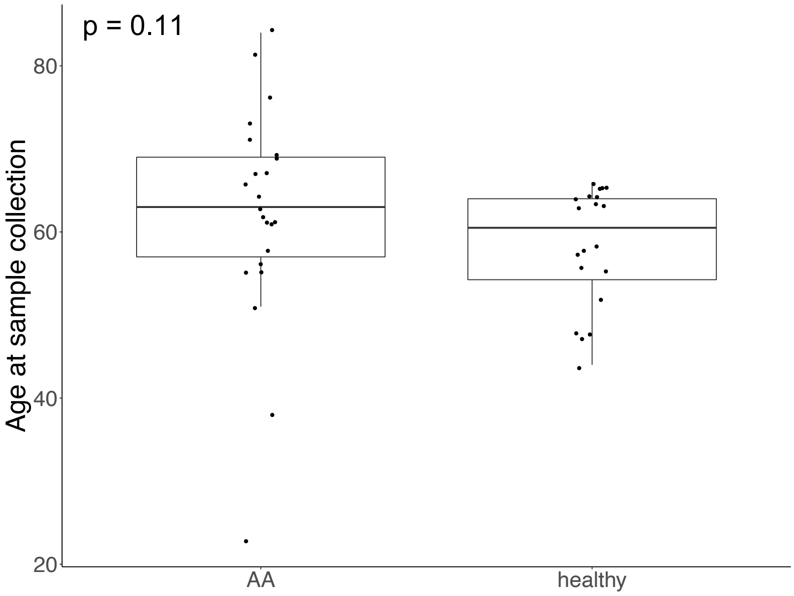

Age in cohorts analyzed with immunogene panel sequencing. Age did not significantly differ between AA and healthy subjects

Figure S7.

A

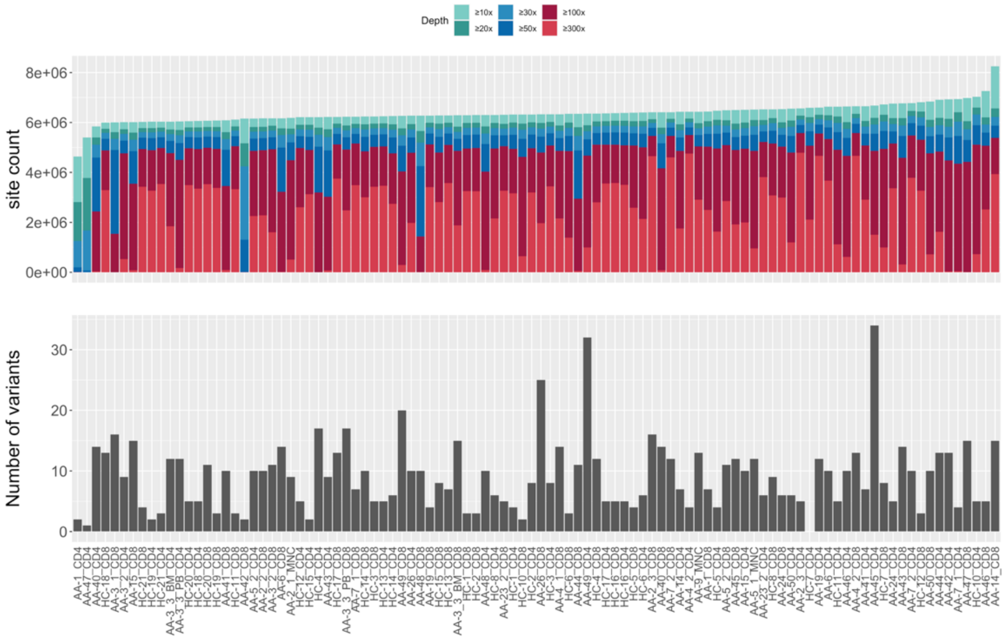

B

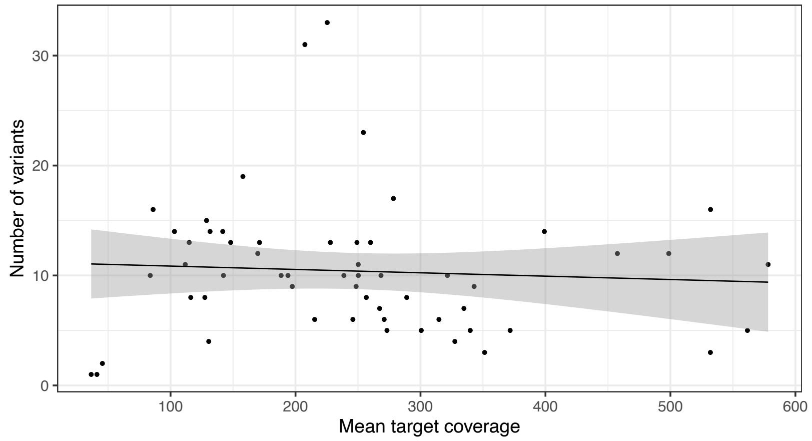

C

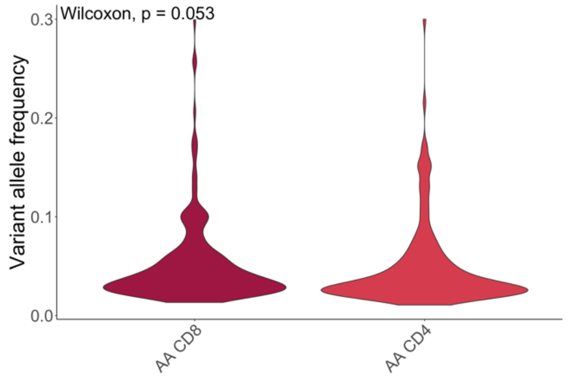

Figure S3 (previous page). Sequencing coverages and variant allele frequencies in immunogenepanel sequencing. (A) In the panel above is shown the number of genomic sites covered >10/20/30/50/100/300x in all samples. In the panel below is number of T cell somatic variants in corresponding sample. (B) Mean target coverage was not associated with number of variants detected with immunogenepanel sequencing (Spearman p = 0.27, $\rho = -0.15$). (C) Variant allele frequencies of fraction-specific variants. The variant allele frequency in AA patients did not significantly differ between CD4+ and CD8+ T cell specific variants.

Figure S4 (next page) All non-synonymous variants of AA patients. Y axis shows the VAF on CD8+ T cells and x axis shows the VAF on CD4+ T cells. LP variants are plotted as triangles and fraction-specific variants as round dots. Variants on JAK-STAT and MAPK signaling pathways are marked with red and blue colours.

Figure S8.

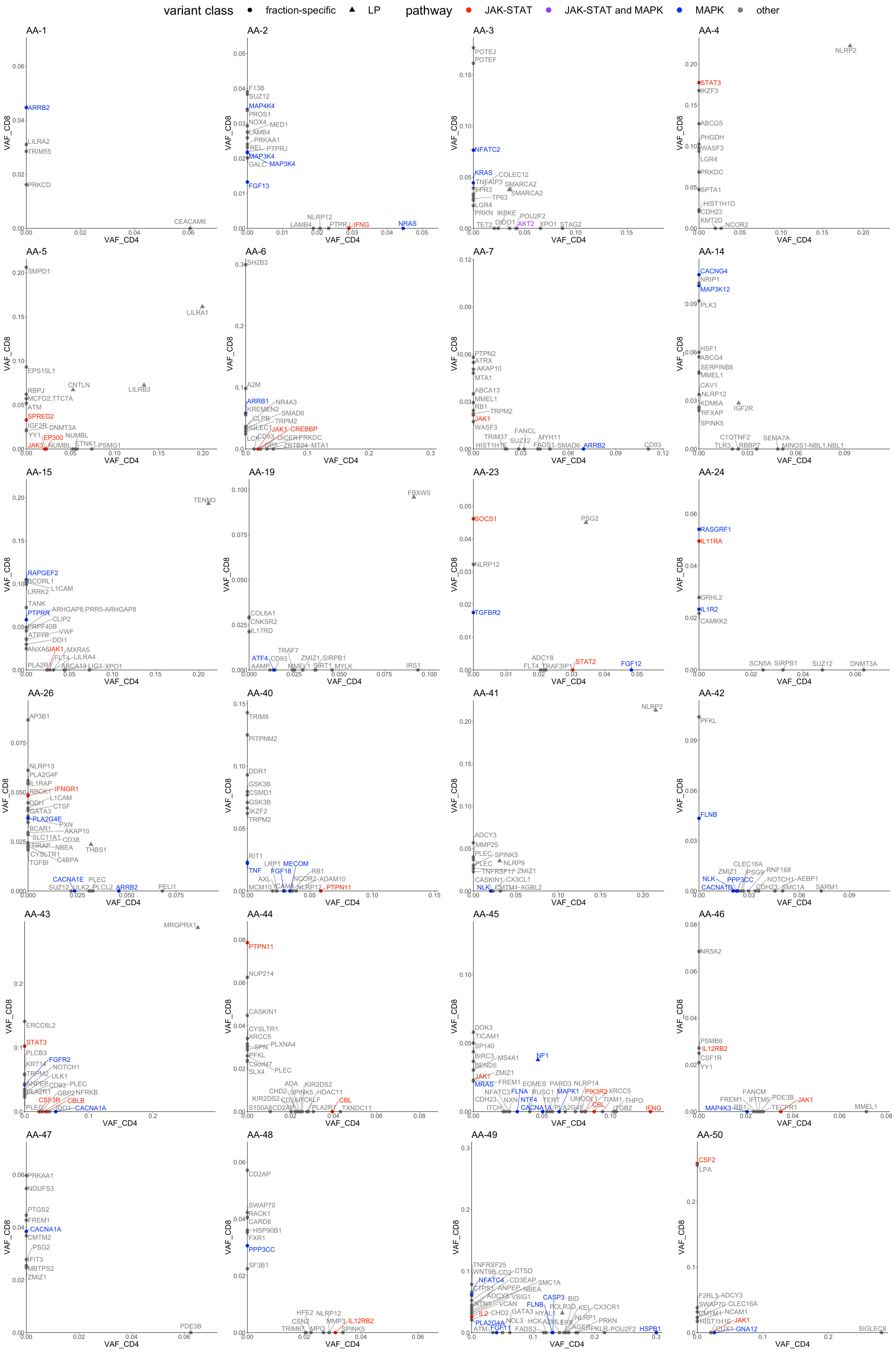
variant class
fraction-specific
LP
pathway
JAK-STAT
JAK-STAT and MAPK
MAPK
other
VAF_CD8
VAF_CD4
AA-1
AA-2
AA-3
AA-4
AA-5
AA-6
AA-7
AA-14
AA-15
AA-19
AA-23
AA-24
AA-26
AA-40
AA-41
AA-42
AA-43
AA-44
AA-45
AA-46
AA-47
AA-48
AA-49
AA-50

Figure S9.

A

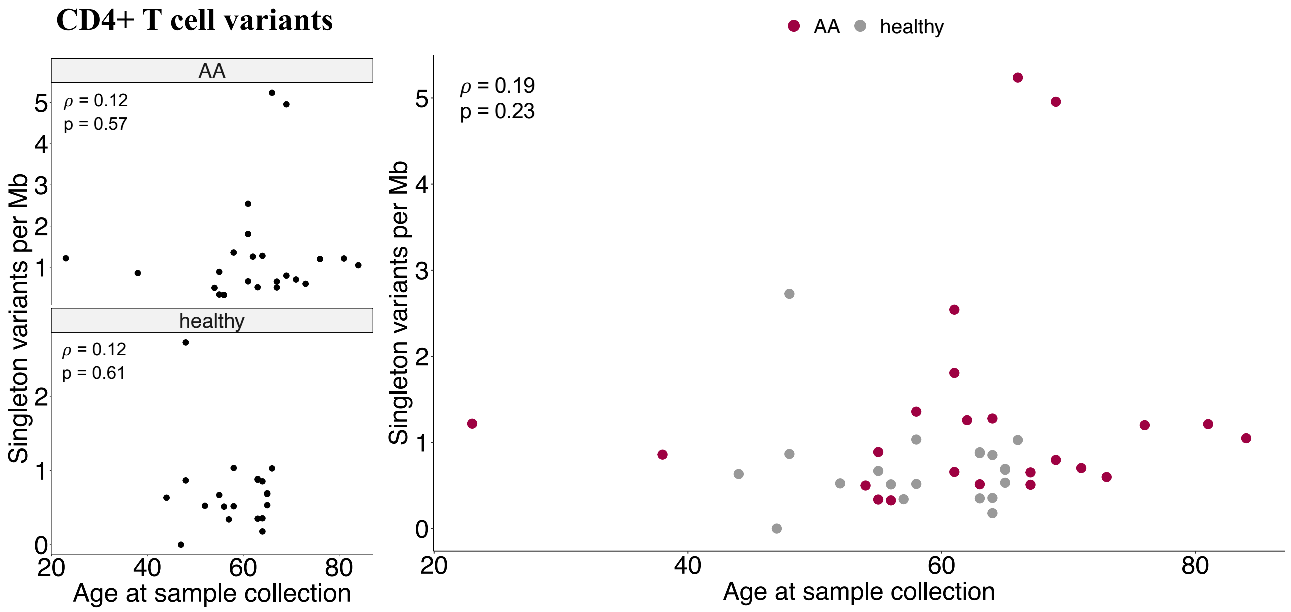

B

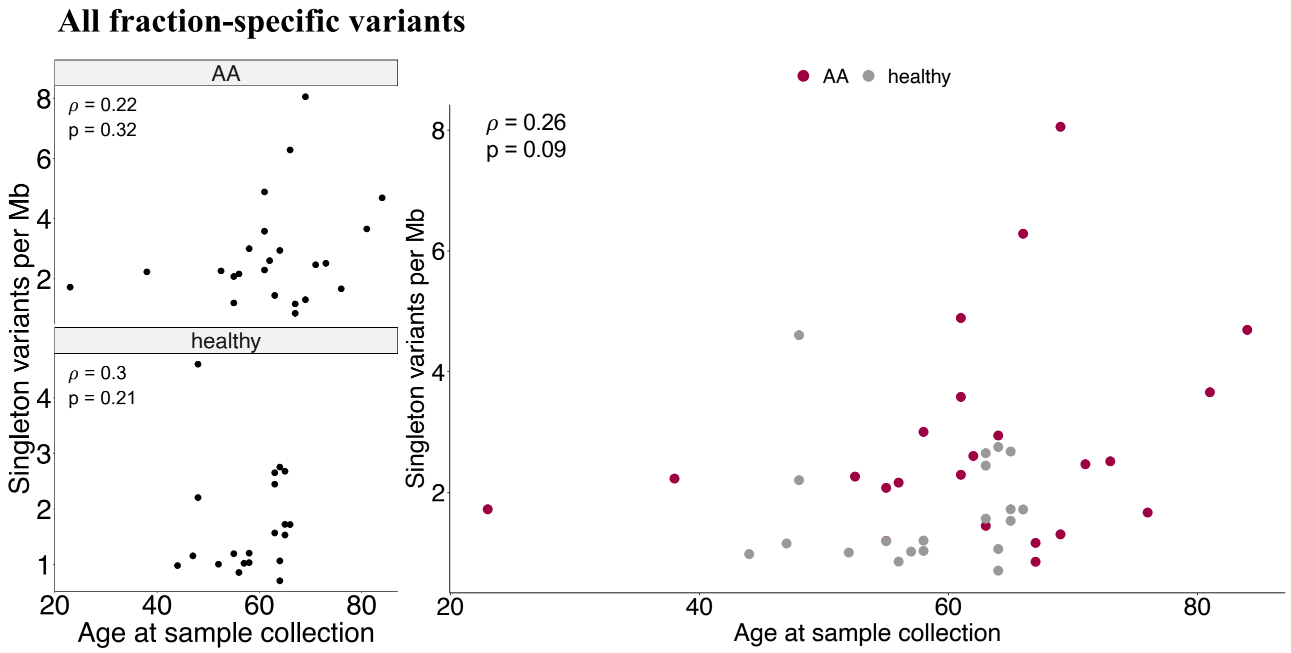

Fraction-specific mutation burden and age. A. CD4+ T cell specific mutation burden and ag**e. B.** On y axis there is the sum of CD4+ and CD8+ T cell mutation burden and on x axis the age at sample collection. In both panels, Spearman test was used to test correlation. Mutation burden was calculated as described in the Supplementary Methods.

Figure S10.

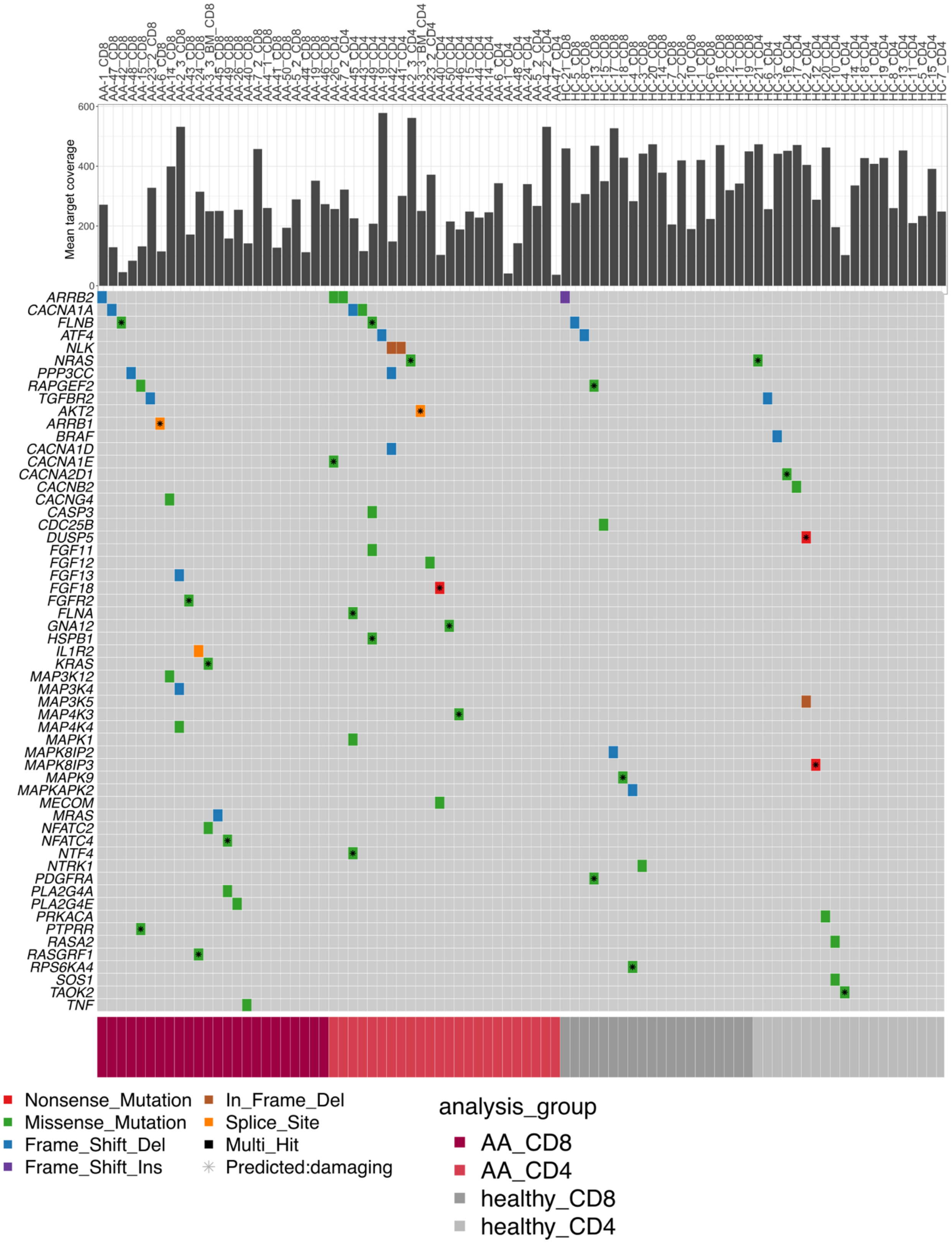

MAPK signaling pathway variants. Variants that were predicted to be damaging are marked with a black asterisk. Mean target coverage of each sample is presented in the top panel.

Figure S11.

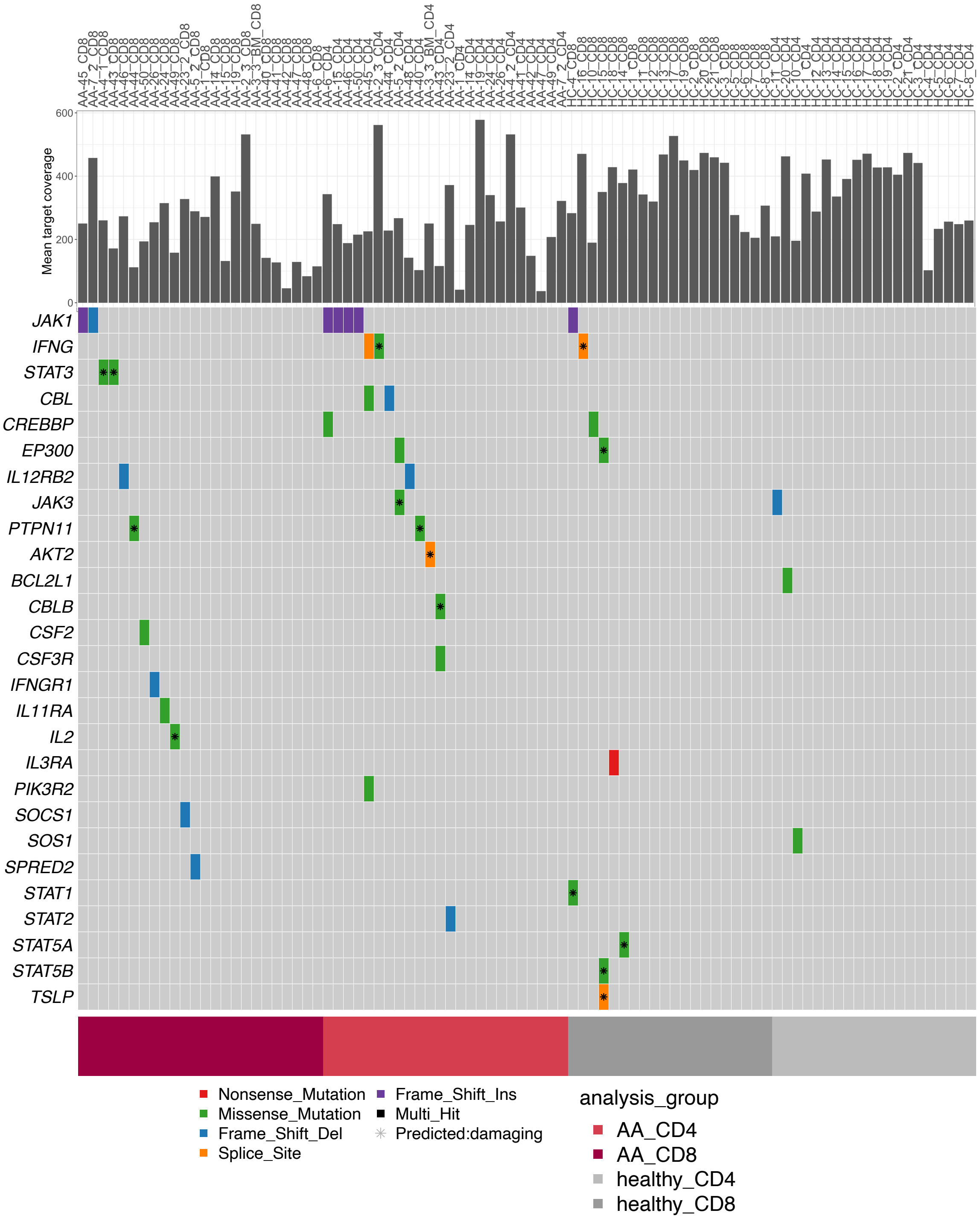

JAK-STAT signaling pathway variants in AA and healthy samples. Variants that were predicted to be damaging are marked with a black asterisk. Mean target coverage of each sample is presented in the top panel.

Figure S12.

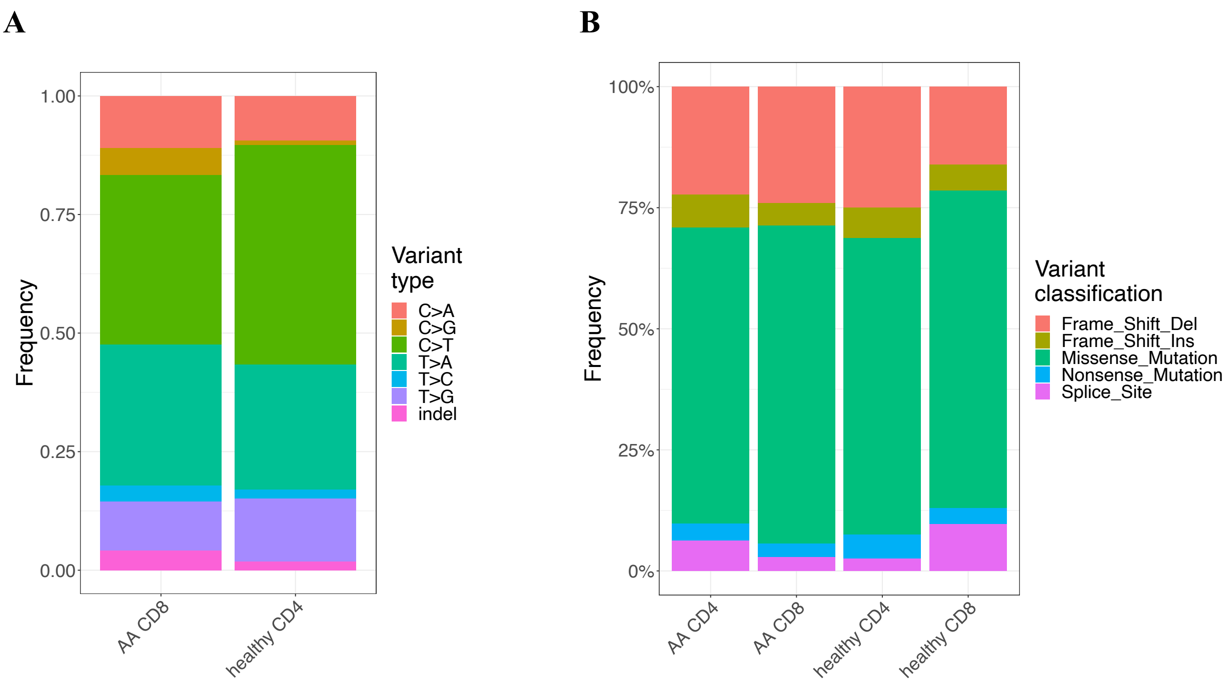

Variant types. (A) SNV variant types from immunogenepanel sequencing. (B) Functional variant types from immunogenepanel sequencing.

Figure S13.

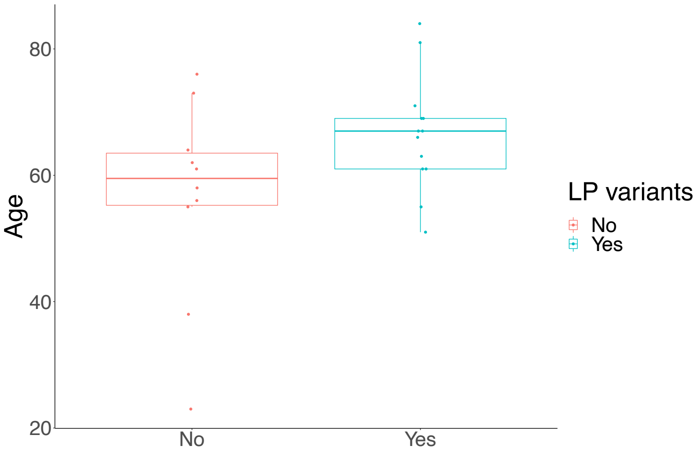

Age in patients with and without LP variants. AA patients with LP variants in immunogene panel sequencing tended to be older than those without LP variants.

Figure S14.

A

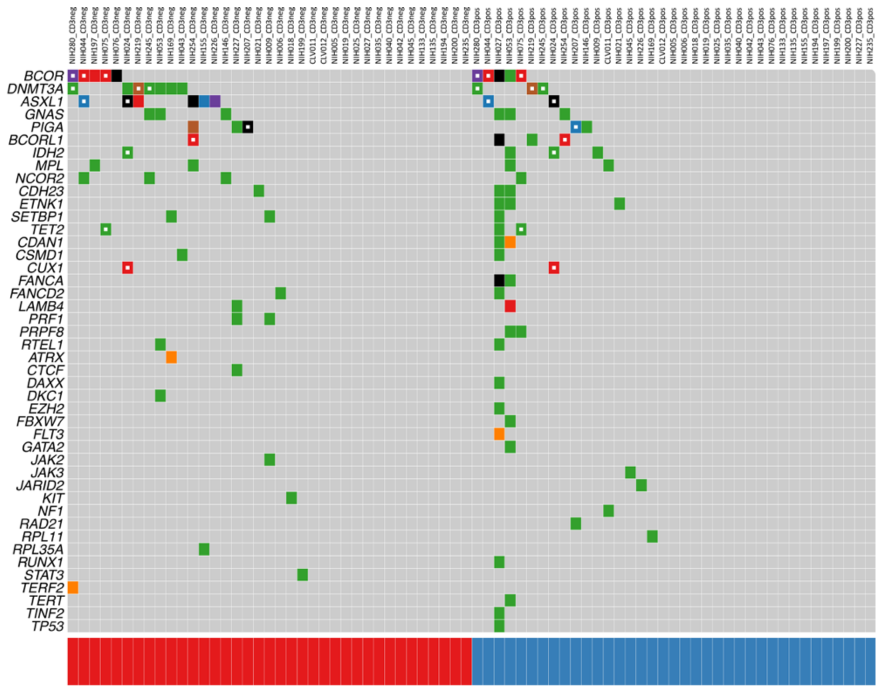

B

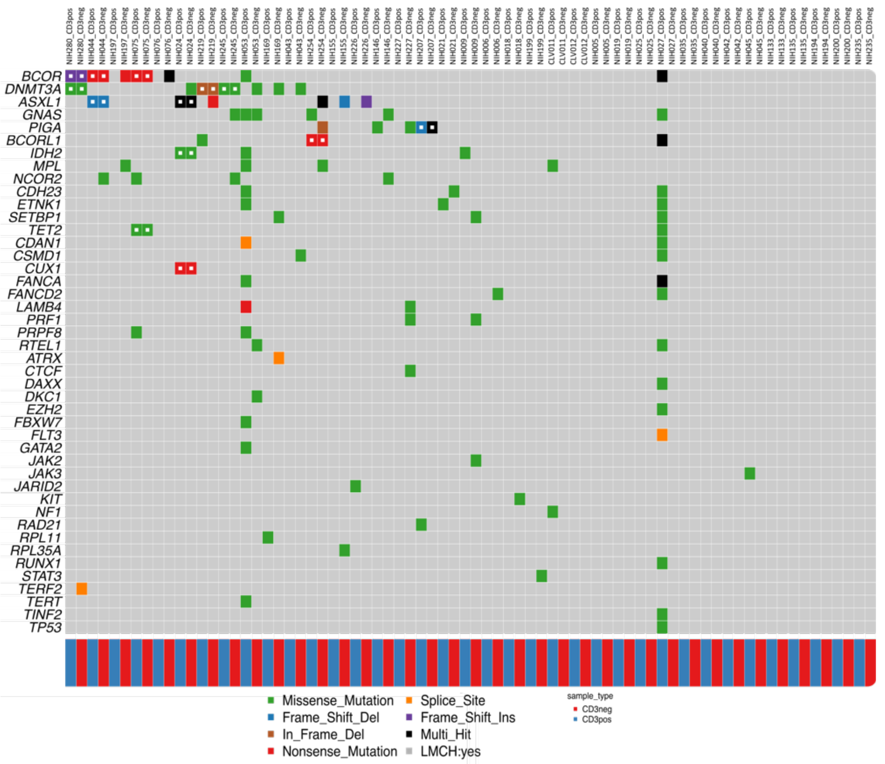

Clonal hematopoiesis variants detected with exome sequencing. Variants in genes related to CH detected with WES of CD3neg and CD3+ MNC of 37 AA patients. Variants detected in both CD3+ and CD3neg MNC in same patient are marked with white squares. In panel A, samples are ordered according to fraction and mutational status and in panel B, CD3+ and CD3neg samples from each patient are shown next to each other.

Table S1. Clinical characteristics of cohorts analyzed by immunogene panel sequencing. (attached as an excel file)

Table S2. Hotspot and STAT3 gain-of-function variants genotyped from AA and healthy samples (attached as an excel file)

Table S3. Variants in immunogene panel sequencing (attached as excel file)

Table S4. Results from amplicon validation (attached as an excel file)

Table S5. Differentially expressed genes in scRNA sequencing used in cluster annotation of AA-4 (attached as an excel file)

Table S6. Differentially expressed genes in scRNA sequencing used in cluster annotation of AA-3 (attached as an excel file)

Table S7. Upregulated genes in TCRBV04-03 clone compared to other CD8+ T_{EMRA} cells in scRNA sequencing

Gene	p value	log of mean fold change
**TRBV4-2**	7,90E-18	5,9
**FTH1**	1,10E-09	1,1
**CSRNP1**	3,70E-07	0,64
**TNFAIP3**	3,80E-07	8,1
**MAFF**	9,40E-07	1,8
**BHLHE40**	1,30E-06	0,99
**ELL2**	1,10E-05	1,5
**TRGV3**	1,90E-05	0,48
**EML4**	2,00E-05	0,51
**NFKBIA**	2,30E-05	2,3
**ZC3H12A**	3,10E-05	0,95
**ZNF644**	4,00E-05	0,64
**MT2A**	4,10E-05	25
**SELL**	4,10E-05	1,1
**GNG2**	4,50E-05	1,1
**ARHGAP15**	7,90E-05	0,93
**SLC2A3**	0,00012	5,2
**FAM177A1**	0,00013	4,2

**NFKB1**	0,00017	0,49
**CD69**	0,00018	8,6
**HSP90AA1**	0,00021	3,3
**RELB**	0,00023	0,58
**RNF19A**	0,00029	0,48
**DSTN**	0,00030	0,46
**FNBP4**	0,00031	0,9
**SRSF5**	0,00033	1
**IL18RAP**	0,00034	0,36
**PDE4B**	0,00035	2,2
**RBM38**	0,00036	0,75
**POLB**	0,00036	0,52
**RAB2A**	0,00056	0,55
**ISG20L2**	0,00061	0,54
**SRSF7**	0,00063	5,4
**IFNGR1**	0,00068	1,1
**MAP3K8**	0,00096	6,3
**MORF4L1**	0,00099	1,5
**TRBC1**	0,0011	0,53
**DNAJA1**	0,0012	2,2
**IRF1**	0,0014	2
**CHD2**	0,0015	0,37
**PTGER4**	0,0017	2,3
**FYN**	0,0017	1,7
**PPM1G**	0,0020	0,36
**PBX4**	0,0021	0,47
**DNTTIP2**	0,0022	0,76
**C1orf52**	0,0025	0,83
**STAT4**	0,0025	0,91
**AKIRIN1**	0,0025	0,36
**RBL2**	0,0025	1,1
**PER1**	0,0025	0,39
**CD320**	0,0027	0,36
**REL**	0,0028	0,42
**SBDS**	0,0028	1,1
**GUK1**	0,0029	0,56
**RAB11B**	0,0034	0,36
**PAXX**	0,0035	0,7

References

1. Savola P, Martelius T, Kankainen M, Huuhtanen J, Lundgren S, Koski Y, et al. Somatic mutations and T-cell clonality in patients with immunodeficiency. Haematologica. Haematologica; 2019 Dec 19;:haematol.2019.220889.

2. Dufva O, Kankainen M, Kelkka T, Sekiguchi N, Awad SA, Eldfors S, et al. Aggressive natural killer-cell leukemia mutational landscape and drug profiling highlight JAK-STAT signaling as therapeutic target. Nat Commun. Nature Publishing Group; 2018 Apr 19;9(1):1567.

3. Adnan Awad S, Kankainen M, Ojala T, Koskenvesa P, Eldfors S, Ghimire B, et al. Mutation accumulation in cancer genes relates to nonoptimal outcome in chronic myeloid leukemia. Blood Adv. 2020 Feb 11;4(3):546–59.

4. Tate JG, Bamford S, Jubb HC, Sondka Z, Beare DM, Bindal N, et al. COSMIC: the Catalogue Of Somatic Mutations In Cancer. Nucleic Acids Res. 2019 Jan 8;47(D1):D941–7.

5. Yoshizato T, Dumitriu B, Hosokawa K, Makishima H, Yoshida K, Townsley D, et al. Somatic Mutations and Clonal Hematopoiesis in Aplastic Anemia. N Engl J Med. 2015 Jul 2;373(1):35–47.

6. Greenplate A, Wang K, Tripathi RM, Palma N, Ali SM, Stephens PJ, et al. Genomic Profiling of T-Cell Neoplasms Reveals Frequent JAK1 and JAK3 Mutations With Clonal Evasion From Targeted Therapies. JCO Precis Oncol. 2018;2018.

7. Li H, Handsaker B, Wysoker A, Fennell T, Ruan J, Homer N, et al. The Sequence Alignment/Map format and SAMtools. Bioinformatics. 2009 Aug 15;25(16):2078–9.

8. Sim N-L, Kumar P, Hu J, Henikoff S, Schneider G, Ng PC. SIFT web server: predicting effects of amino acid substitutions on proteins. Nucleic Acids Res. 2012 Jul;40(Web Server issue):W452–7.

9. Adzhubei I, Jordan DM, Sunyaev SR. Predicting functional effect of human missense mutations using PolyPhen-2. Curr Protoc Hum Genet. John Wiley & Sons, Ltd; 2013 Jan;Chapter 7(1):Unit7.20–7.20.41.

10. Chun S, Fay JC. Identification of deleterious mutations within three human genomes. Genome Res. Cold Spring Harbor Lab; 2009 Sep;19(9):1553–61.

11. Schwarz JM, Cooper DN, Schuelke M, Seelow D. MutationTaster2: mutation prediction for the deep-sequencing age. Nat Methods. Nature Publishing Group; 2014 Apr;11(4):361–2.

12. Reva B, Antipin Y, Sander C. Predicting the functional impact of protein mutations: application to cancer genomics. Nucleic Acids Res. 2011 Sep 1;39(17):e118.

13. Shihab HA, Gough J, Cooper DN, Stenson PD, Barker GLA, Edwards KJ, et al. Predicting the functional, molecular, and phenotypic consequences of amino acid substitutions using hidden Markov models. Hum Mutat. John Wiley & Sons, Ltd; 2013 Jan;34(1):57–65.

14. Carter H, Douville C, Stenson PD, Cooper DN, Karchin R. Identifying Mendelian disease genes with the variant effect scoring tool. BMC Genomics. BioMed Central; 2013;14 Suppl 3(3):S3–16.

15. Douville C, Masica DL, Stenson PD, Cooper DN, Gygax DM, Kim R, et al. Assessing the Pathogenicity of Insertion and Deletion Variants with the Variant Effect Scoring Tool (VEST-Indel). Hum Mutat. John Wiley & Sons, Ltd; 2016 Jan;37(1):28–35.

16. Kircher M, Witten DM, Jain P, O'Roak BJ, Cooper GM, Shendure J. A general framework for estimating the relative pathogenicity of human genetic variants. Nat Genet. Nature Publishing Group; 2014 Mar;46(3):310–5.

17. Rentzsch P, Witten D, Cooper GM, Shendure J, Kircher M. CADD: predicting the deleteriousness of variants throughout the human genome. Nucleic Acids Res. 2019 Jan 8;47(D1):D886–94.

18. Garber M, Guttman M, Clamp M, Zody MC, Friedman N, Xie X. Identifying novel constrained elements by exploiting biased substitution patterns. Bioinformatics. 2009 Jun 15;25(12):i54–62.

19. Pollard KS, Hubisz MJ, Rosenbloom KR, Siepel A. Detection of nonneutral substitution rates on mammalian phylogenies. Genome Res. Cold Spring Harbor Lab; 2010 Jan;20(1):110–21.

20. Dong C, Wei P, Jian X, Gibbs R, Boerwinkle E, Wang K, et al. Comparison and integration of deleteriousness prediction methods for nonsynonymous SNVs in whole exome sequencing studies. Hum Mol Genet. 2015 Apr 15;24(8):2125–37.

21. Savola P, Kelkka T, Rajala HL, Kuuliala A, Kuuliala K, Eldfors S, et al. Somatic mutations in clonally expanded cytotoxic T lymphocytes in patients with newly diagnosed rheumatoid arthritis. Nat Commun. Nature Publishing Group; 2017 Jun 21;8:15869.

22. Lopez R, Regier J, Cole MB, Jordan MI, Yosef N. Deep generative modeling for single-cell transcriptomics. Nat Methods. Nature Publishing Group; 2018 Dec;15(12):1053–8.

23. Soneson C, Robinson MD. Bias, robustness and scalability in single-cell differential expression analysis. Nat Methods. Nature Publishing Group; 2018 Apr;15(4):255–61.

24. Blom S, Paavolainen L, Bychkov D, Turkki R, Mäki-Teeri P, Hemmes A, et al. Systems pathology by multiplexed immunohistochemistry and whole-slide digital image analysis. Sci Rep. Nature Publishing Group; 2017 Nov 14;7(1):15580–13.

25. Brück O, Blom S, Dufva O, Turkki R, Chheda H, Ribeiro A, et al. Immune cell contexture in the bone marrow tumor microenvironment impacts therapy response in CML. Leukemia. Nature Publishing Group; 2018 Jul;32(7):1643–56.

26. Carpenter AE, Jones TR, Lamprecht MR, Clarke C, Kang IH, Friman O, et al. CellProfiler: image analysis software for identifying and quantifying cell phenotypes. Genome Biol. BioMed Central; 2006;7(10):R100–11.

27. Zhang L, Yu X, Zheng L, Zhang Y, Li Y, Fang Q, et al. Lineage tracking reveals dynamic relationships of T cells in colorectal cancer. Nature. Nature Publishing Group; 2018 Dec;564(7735):268–72.
